# Notre-Dame de Paris: The first iron lady? Archaeometallurgical study and dating of the Parisian cathedral iron reinforcements

**DOI:** 10.1371/journal.pone.0280945

**Published:** 2023-03-15

**Authors:** Maxime L’Héritier, Aurélia Azéma, Delphine Syvilay, Emmanuelle Delqué-Kolic, Lucile Beck, Ivan Guillot, Mathilde Bernard, Philippe Dillmann

**Affiliations:** 1 Archéologie et Sciences de l’Antiquité, ArScAn CNRS UMR7041, Université Paris 8 Vincennes-Saint-Denis, Saint-Denis, France; 2 Laboratoire de Recherche des Monuments Historiques LRMH, CRC UAR 3224, Muséum National d’Histoire Naturelle MHNH—CNRS—Ministère de la Culture, Champs-sur-Marne, France; 3 Sorbonne University Abu Dhabi, Abu Dhabi, United Arab Emirates; 4 Laboratoire de Mesure du Carbone 14 (LMC14), LSCE/IPSL, CEA-CNRS-UVSQ, Université Paris-Saclay, Gif-sur-Yvette, France; 5 CNRS, ICMPE (Institut de Chimie et des Matériaux Paris-Est), UMR 7182, Université Paris-Est Créteil, Thiais, France; 6 Laboratoire Archéomatériaux et Prévision de l’Altération, LAPA: LMC IRAMAT UMR7065 CNRS et NIMBE UMR3685 CEA/CNRS, Université Paris Saclay, CEA Saclay, Gif sur Yvette cedex, France; University of California Santa Cruz, UNITED STATES

## Abstract

The study of iron reinforcements used in the construction of Notre-Dame de Paris offers a glimpse into the innovation that took place on this building site in the mid-12^th^ century, adapting metal to create a novel architecture. The restoration of the monument after the 2019 fire offered unique possibilities to investigate its iron armatures and to sample 12 iron staples from different locations (tribunes, nave aisles and upper walls). Six of them were dated thanks to the development of an innovative methodology based on radiocarbon dating. They reveal that Notre-Dame is the first known Gothic cathedral where iron was massively used as a proper construction material to bind stones throughout its entire construction, leading to a better understanding of the master masons’ thinking. Moreover, a metallographic study and slag inclusion chemical analyses of the staples provide the first study of iron supply for a great medieval Parisian building yard, renewing our understanding of iron circulation, trade and forging in the 12^th^ and 13^th^ century capital of the French kingdom. The highlighting of numerous welds in all iron staples and the multiple provenances sheds light on the activity of the iron market in this major medieval European city and the nature of the goods that circulated, and questions the possible importance of recycling.

## Introduction

Notre-Dame de Paris is an unprecedented monumental work in the history of Gothic and Christian architecture. With its vaults peaking at 32m high, the Parisian cathedral was the tallest building ever built at that time [1], even surpassing the greatest Ottonian or Romanesque achievements, such as the abbey church of Cluny (29.50 m). With its five-vessel nave, measuring nearly 37 m wide and 125 m long, Notre-Dame covers a considerable area, but it mostly impresses in terms of height: at the same period, the cathedrals of Laon, Senlis and Sens barely reached 24 m under the vault and only 22 m at Noyon. No comparable monument was built until the beginning of the 13^th^ century, maybe two or three generations of builders later, with the cathedrals of Chartres, Bourges, Reims, Amiens or Beauvais, finally exceeding the height of Notre-Dame’s vaults.

Notre-Dame’s elevation was made possible by the synthesis of innovative technical procedures devised by the first architect of Notre-Dame. The use of the ribbed crossing, mastered since the end of the 11^th^ century, as well as the construction of very thin vaults (15 to 30 cm thick), made it possible to lighten the structure and to create more openings, with the weight of the vaults resting solely on the piers [2]. The five-vessel plan, uncommon at the time, offered a wider base to sustain this unusual elevation. In the four-storey elevation, a level of tribunes recalls the techniques used in Romanesque buildings to support the upper parts of the nave and limits the height of the great arches. Higher up, the 12^th^-century bays surmounting roses are of small dimensions and are pierced in an entirely masonry wall. Finally, on the outside, slender flying buttresses contain the thrusts of the upper parts of the building; a technique only experimented before in Saint-Germain-des-Prés, Domont, Saint-Quiriace de Provins or Saint-Maclou de Pontoise [3, 4]. However, Notre-Dame’s flying buttresses have a double arch and are much thinner than these other examples and their origin was long questioned, especially for the choir. Laser scanning surveys eventually enabled the deformations of the building since its elevation to be measured and confirmed the authenticity of the flying buttresses [5, 6].

All these architectural feats were indeed crucial for the construction of Notre-Dame to succeed. But were they sufficient? The contribution of the types of materials was never really addressed among the construction techniques [7]. They are recorded merely as lists in the remaining folios of the archival record books, even if sometimes the reason for the choice made is succinctly described, most of time about the stones (proximity of the quarry, color and workability) and for late medieval or modern periods for example [8, 9]. Nevertheless, the material properties clearly determine the structure’s stability. The implementation of iron and its functions in the initial design of several 13^th^ century cathedrals is now well known [10, 11], but has never been explored this far in the case of Notre-Dame. As in all these monuments, iron might also have played a major role in the cathedral’s elevation from its earliest construction campaigns. This paper aims to address this specific question using the interdisciplinary background built over the past two decades [12]. The monument currently in restoration offers unique access to places in the cathedral that were unattainable until now, providing the opportunity to detect for the first time the use of iron reinforcements in the cathedral.

The use of iron armatures was long considered as a feature of late restoration (usually 19^th^ c.) and was therefore rarely studied in the past. Up to the 1980s, following this opinion, certain monuments bearing such iron reinforcements were mutilated, such as Beauvais cathedral where iron tie-rods were removed, leading to major structural issues for the monument [13, 14]. So far, the earliest Gothic monuments in which iron armatures have been discovered were built in the beginning of the 13^th^ c. (Chartres, Bourges, Reims…) [12, 15, 16] or in the late 12^th^ century, with the peculiar example of the southern transept of Soissons cathedral [17]. In mid-12^th^ cathedrals such as Laon and Noyon, only small iron pins were sometimes used inside some of the columns [18]. Yet, even in recent studies [19], the case of Notre-Dame de Paris has never been properly addressed beyond Viollet-le-Duc’s remarks in his *Dictionary of Architecture*, claiming that some staples were placed inside the choir’s checked cornice in 1195 [20, tome 2, article chaînage].

The fire that struck Notre-Dame de Paris in April 2019 shed light on unprecedented sets of iron staples used in several parts of the building. Were they implemented in the initial phases of construction or added during the modifications of the 13^th^ century or even later, during restoration work such as that commissioned in the 18^th^ c. by the Cardinal de Noailles or conducted in the 19^th^ c. by Viollet-le-Duc? The recent development of an innovative methodology, based on an extensive characterization of the metal combined with an original application of the radiocarbon method allows us to directly date iron armatures in order to determine whether they were used for construction or consolidation purposes [10]. Its application to the study of the reinforcements of Notre-Dame de Paris will situate them in the chronology of the cathedral and shed new light on the beginnings of Gothic construction, leading to a better understanding of the master masons’ thinking. Furthermore, one cannot understand ancient materials without considering their quality or supply. It is indeed crucial to understand the production and circulation of iron towards one of the major medieval building yards in the biggest European city over the long time scale as well as to qualify the work of the smiths who shaped the iron reinforcements for the construction site.

## Materials & methods

### Prospection in Notre-Dame

**Fig 1:**
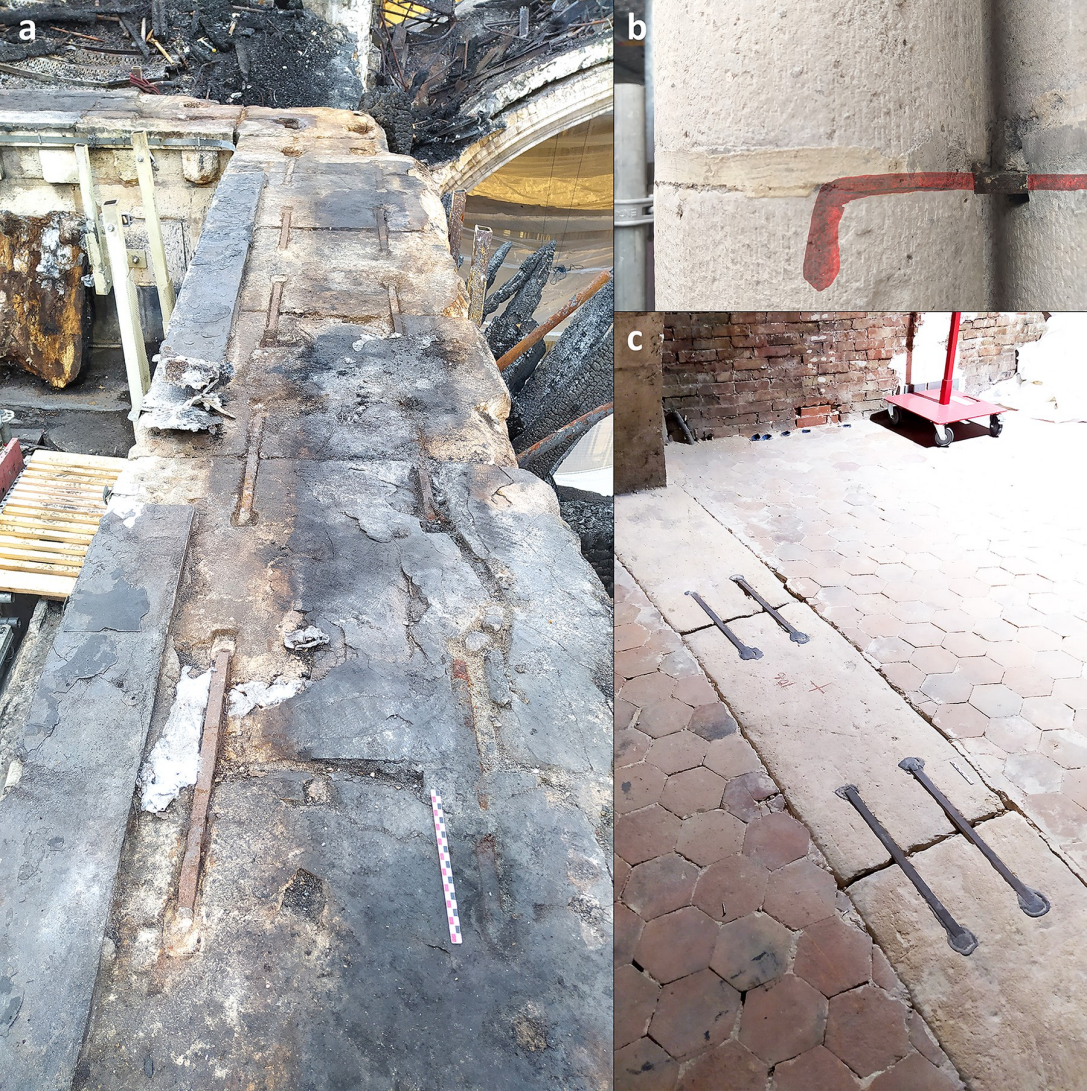
Iron armatures in Notre-Dame. a. Iron staples of the top walls (southern nave looking on the angle of the southern transept). b. Iron staples inside the monolithic columns of the nave (view of the staple in red). c. Iron staples in the tribunes of the choir (staples n° 121 to 124).

Extensive prospection was carried out in Notre-Dame following the 2019 fire to investigate the use of iron reinforcements in the masonry. Access to the upper parts of the building was made possible thanks to harnessing and scaffolding. Some parts of the upper walls, located under the provisional roof in the choir could not be accessed. No geophysical means of detection could be performed in the upper masonries due to lack of access from the exterior of the building. The result of these first prospections shows that iron staples were used at different levels in Notre-Dame’s structure (Fig 1).

**Fig 2:**
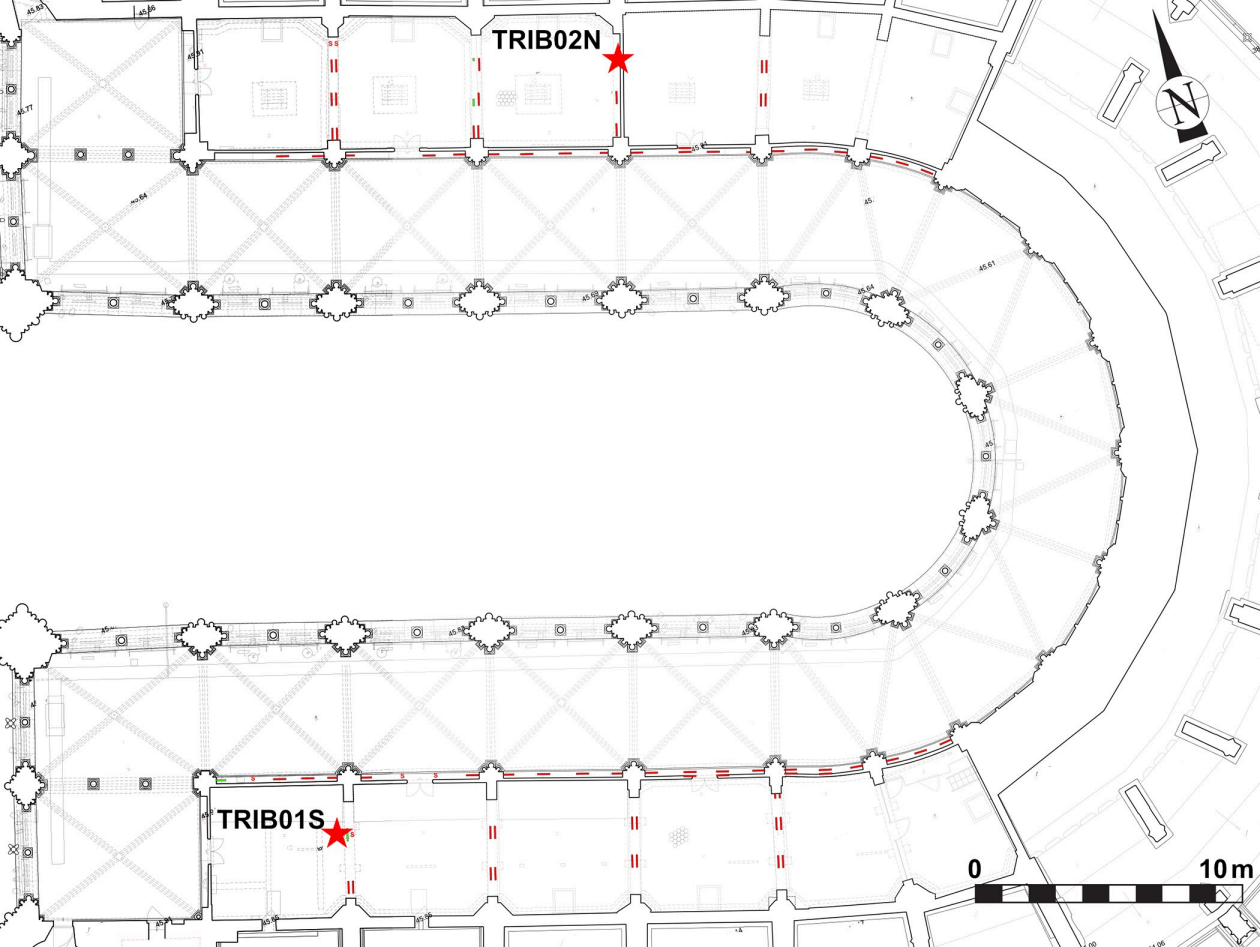
Map of the tribunes of the choir, showing the location of the staples (red lines) and the sampling (red stars).

The lowest set of staples is located in the tribunes, which are the second level of elevation above the arches. Two rows of staples can be found on the floor of the tribunes (Fig 2), on the upper surface of the arches situated between the inner and outer aisles (in the nave) or ambulatories (in the choir) and also above the transverse arches of the outer aisle (or ambulatory). None were found in the transept. A total of 170 staples were listed in the nave and choir. However, many staples of the same set are embedded in the masonry, either below the outer walls of the tribunes or below renovated parts of the floor, and are therefore not visible. The number of staples can be roughly estimated at about 300 to 400 (about 200 in the nave and 150 in the choir).

In the nave, staples also systematically attach the blocks that form the monolithic columns decorating the chapels’ walls (Fig 1). Each of the three 8 m high columns circling each pier is made of two to three blocks of stone, whose upper part is joined to the wall using perpendicular staples. The same feature is used for the piers situated in between the inner and outer aisles, which are decorated with several monolithic columns in at least 5 or 6 pieces about 16 m high leaning against the piers. The systematic use of staples was revealed using a metal detector.

**Fig 3:**
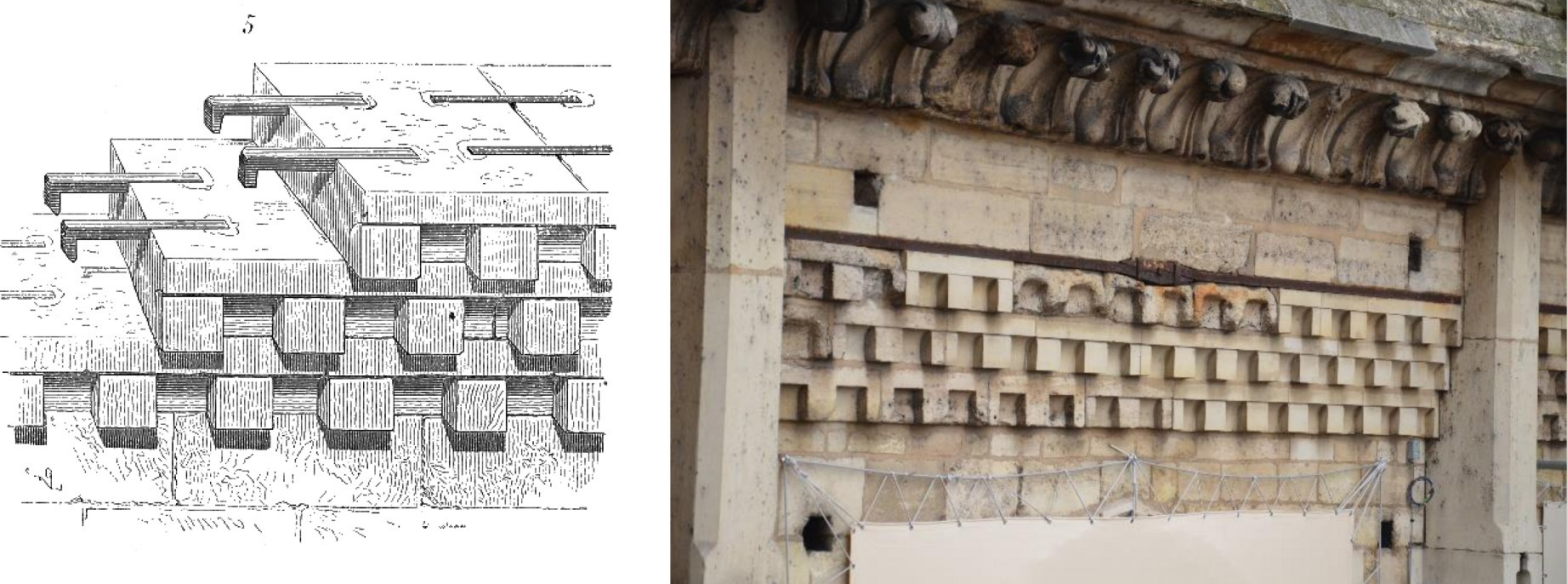
View of the checked cornice of the choir with its series of staples drawn by Viollet-le-Duc (left) [19 tome 2, p. 400] and today, with the chains implemented by Lassus (right).

**Fig 4:**
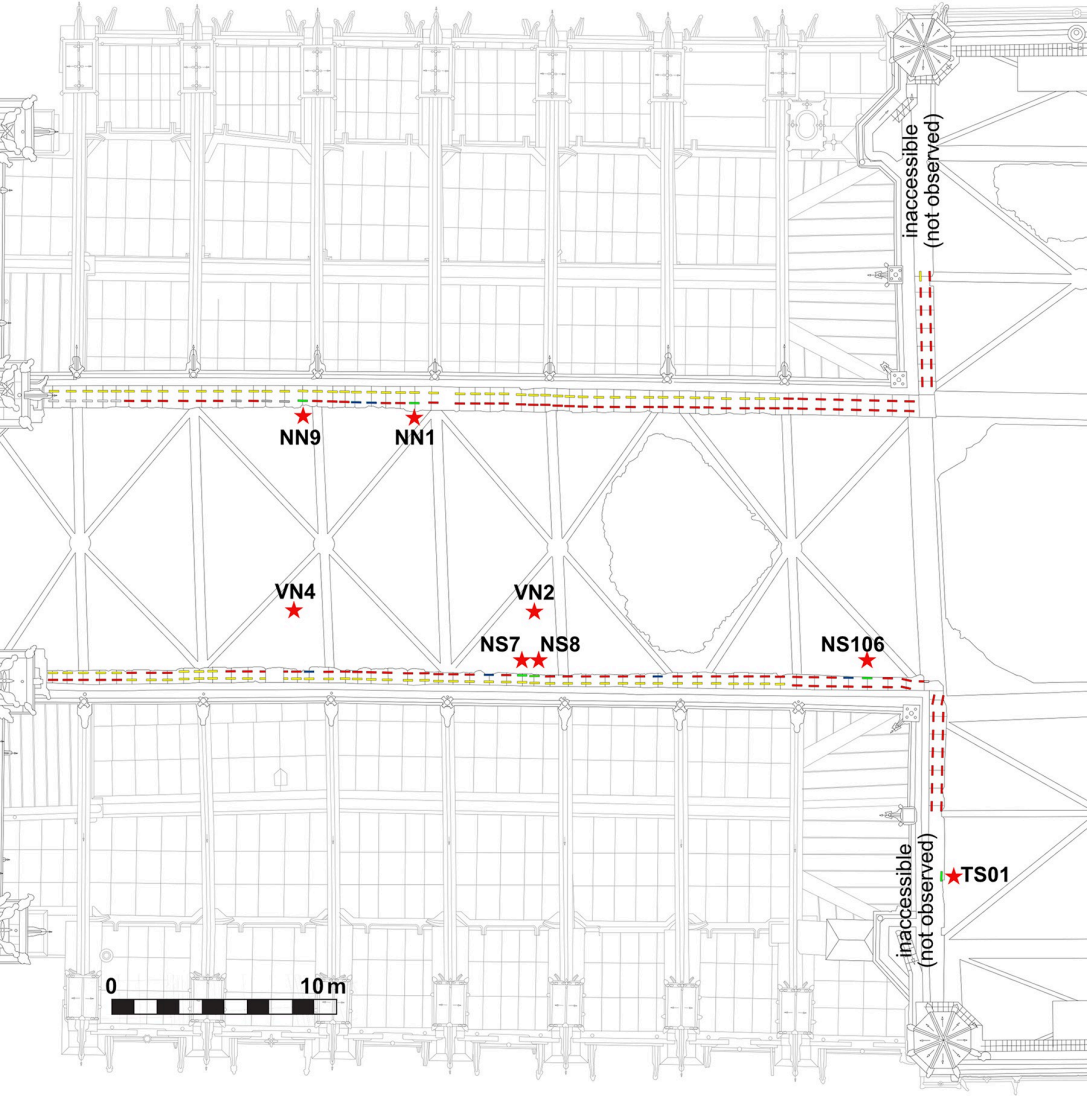
Map of the upper walls in the nave, showing the location of the staples and the sampling (Red = staples observed, Yellow, staples not measured (below provisional roof beams), blue = staples that fell with the fire, Green = staples sampled, Grey = staples formerly removed). Red stars: sampling locations.

**Fig 5:**
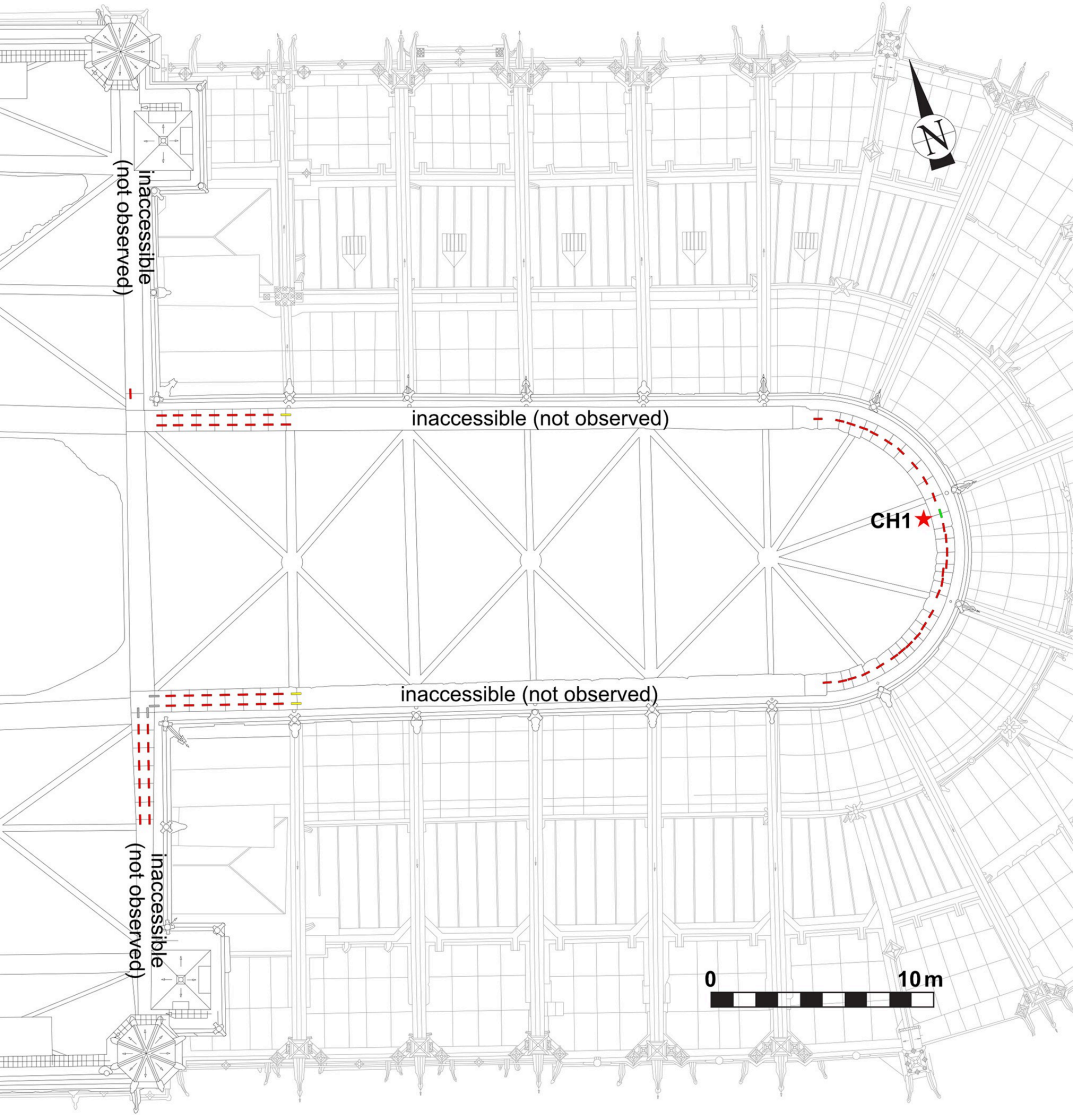
Map of the upper walls in the choir, showing the location of the staples and the sampling (Red = staples observed, Yellow, staples not measured (below provisional roof beams), blue = staples that fell with the fire, Green = staples sampled, Grey = staples formerly removed). Red stars: sampling locations.

The checked cornice (Fig 3) described by Viollet-le-Duc could not be studied as no staples are visible without dismantling the masonry. However, an unknown set of staples was discovered on the top of the upper walls (Figs 4 & 5), just below the timbers of the burnt framework. As in the tribunes and in the checked cornice (according to Viollet-le-Duc’s drawings, Fig 3), the system again shows two rows of staples in the nave and in the bays adjacent to the transept crossing. In the apse however, there is only one row of staples. Due to lack of accessibility, the system could not be observed in the middle bays of the choir or in the northern and southern bays of the transepts. More than 200 staples were listed, with an estimated 500 to 600 overall.

Other reinforcements clearly belonging to the mid-19^th^ century restoration campaigns such as iron chains and tie-rods in the top walls of the choir and above its upper vaults were also observed [21] (Fig 3). They will however not be considered in the present paper.

Iron reinforcements therefore seem to have been used extensively in Notre-Dame’s construction in its entire height. Moreover, the sampling possibilities, which gave access to entire staples that were removed and will be replaced, is a particular feature of this study compared to most previous ones made on other monuments [11, 22, 23].

### Sampling

All accessible iron armatures were mapped and measured: about 170 staples for the upper walls and 110 for the tribunes (Figs 2, 4 & 5). Sampling for metallographic analyses was performed according to several conservation criteria on available material, already broken or degraded by the fire (Table 1). Authorisations for sampling were given by the Direction Régionale des Affaires Culturelles (DRAC Ile-de-France) and by the public institution for the restoration of Notre-Dame (Rebâtir Notre-Dame de Paris, RNDP). All necessary permits were obtained for the described study, which complied with all relevant regulations.

**Table 1:** List of studied artefacts and samples.

Artefact reference	Type	Location	Number of cross sections (T = transverse, L = longitudinal)
NN1	staple	Upper walls (nave north)	3 (1T + 2L)
NN9	staple	Upper walls (nave north)	3 (1T + 2L)
NS7	staple	Upper walls (nave south)	1 (1T)
NS8	staple	Upper walls (nave south)	2 (1T + 1L)
NS106	staple	Upper walls (nave south)	2 (2T)
VN2	staple	Upper walls (nave south)	1 (1T)
VN4	staple	Upper walls (nave south)	2 (1T + 2L)
CH1	staple	Upper walls (nave south)	3 (1T + 2L)
TS01	staple	Upper walls (southern transept)	2 (2T)
GUA01	staple	Nave aisle (Guadalupe chapel)	1 (1L)
TRIB01S	staple	Tribunes (choir south)	1 (1L)
TRIB02N	staple	Tribunes (choir north)	1 (1L)
**Total**	**12**		**22**

**Fig 6:**
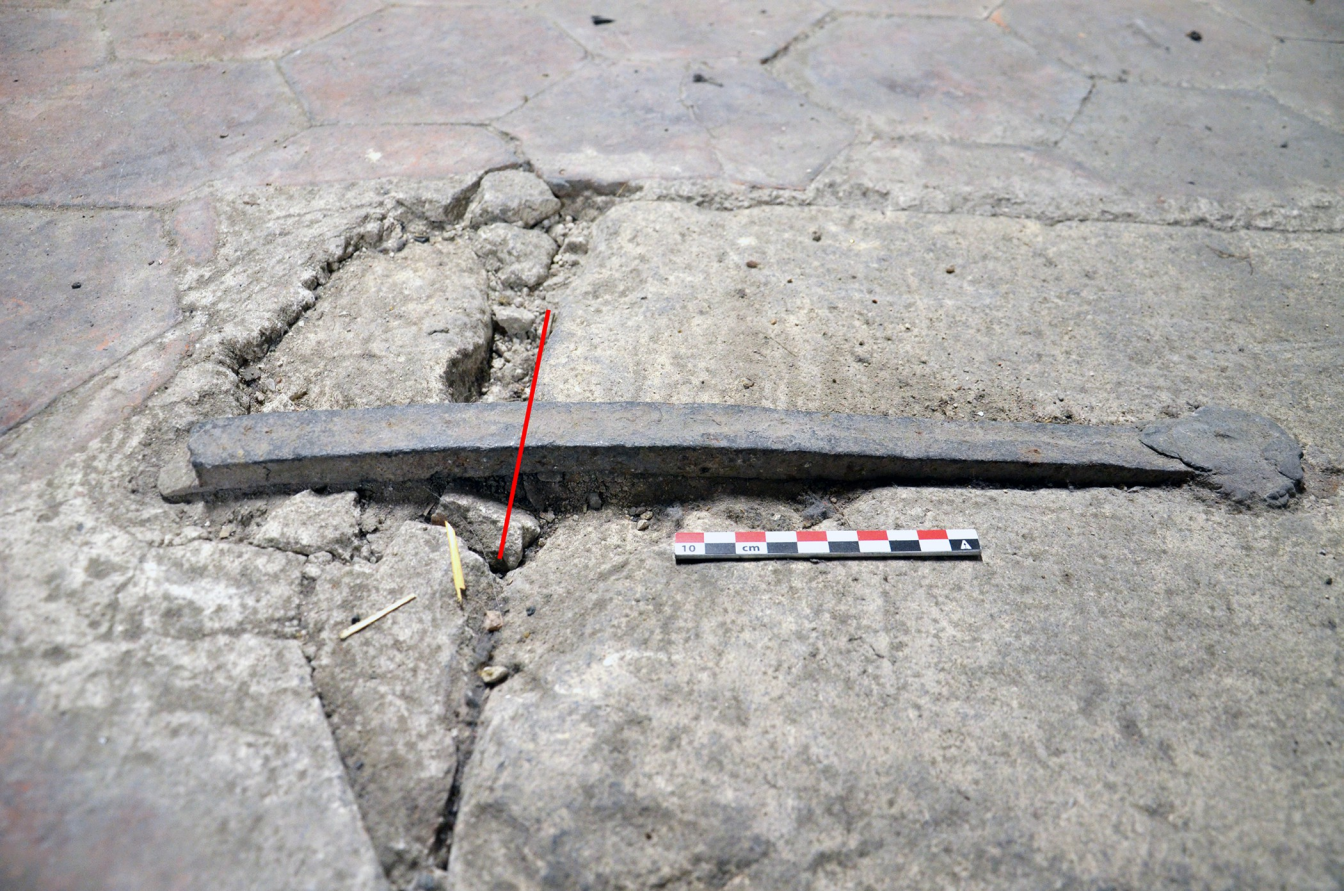
Broken staple in the tribunes (TRIB01S). The red line indicates the sampling. The sample was then cut longitudinally for analysis.

**Fig 7:**
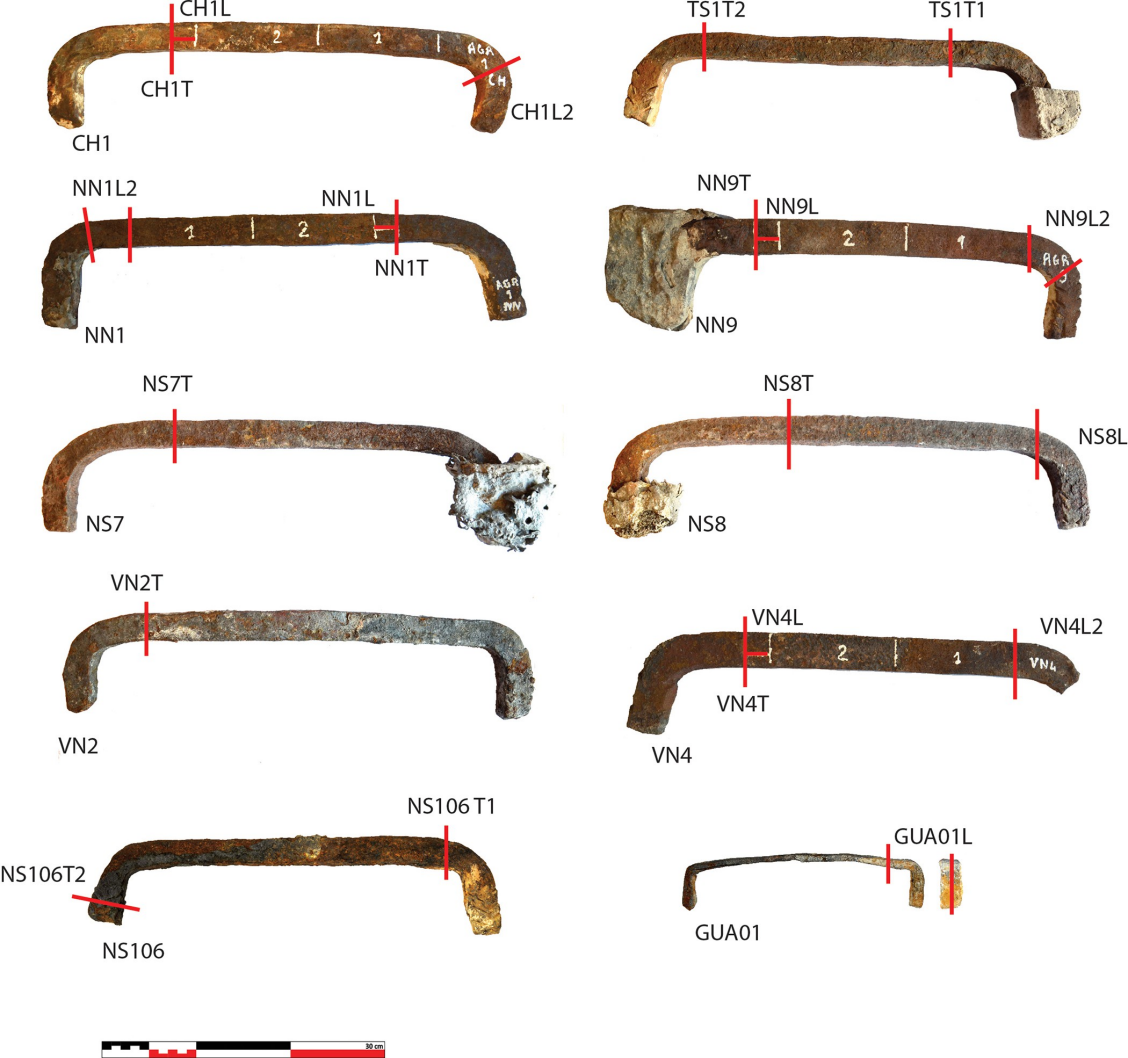
Sampling on the studied staples.

In the tribunes where the masonry was not damaged by the blaze and most of the staples are intact, only two staples from the choir that had already been broken in previous arrangements of the tribune floor could be partially sampled (TRIB01S & 02N) (Fig 6). One staple was dismantled during the restoration of the Notre-Dame of Guadalupe chapel in the northern side of the nave as the monolithic column it was attaching was partly broken and needed to be replaced (GUA01). In the upper parts, two entire staples, which fell down during the fire, were found in the archaeological excavation of the burnt remains on the vaults (VN2 & VN4). The archaeological grid [24] that was set up for this excavation enabled to identify their original position during the archaeological prospections, on the southern nave wall. Seven other staples still in place were removed in full for study with the authorisation of the architects and contracting authority (RNDP): five in the North and South sides of the nave (NN1 & 9, NS7 & 8 & 106), one in the southern transept (TS01) and one in the choir (CH1), in compliance with the possibilities granted (staples joining fractured blocks of stone). On most staples, several cross sections were cut in order to investigate the heterogeneity of the material (Fig 7).

## Methods

### Metallographic analyses

Metallographic examinations were carried out on all samples using Nital etching on polished (SiC papers grade 80 to 1200, diamond paste 9, 3 and 1 μm) cross sections to reveal carburised zones (presence of carbides) [25], as well as Oberhoffer’s reagent on a selection of sections to highlight the distribution of phosphorus [26]. Numerous micrometric and millimetric slag inclusions (SI) entrapped within the metallic matrix were also observed. The presence of such SI is typical of ancient ferrous alloys and is linked to the ancient ore smelting processes: iron was never fully produced in a liquid state before the development of the Bessemer and Thomas refining processes in the 19^th^ century (the refining stage partly took place in a solid state before that). This is especially the case for iron produced in bloomery furnaces during the Middle Ages: SI come from the compounds of the ore which are not completely reduced during smelting and form the slag, which is not fully separated from the iron bloom. Hence, SI are mostly composed of iron silicates or fayalite (Fe_2_SiO_4_) and iron oxides (FeO) alongside with other oxides (Al_2_O_3_, P_2_O_5_, K_2_O, CaO, MnO…). Moreover, within their composition these SI also potentially bear the signature of the original ore as well as the imprint of the metallurgical and forging processes that were used. In the case of bloomery iron, the chemical analysis of SI has been used for provenance purposes [27–29].

### SI analyses and data treatment

SI were analysed and treated according to the protocol proposed by Dillmann and L’Héritier [30] and adapted by Disser et al [31]. To enhance the representativeness of the average slag produced and to obtain information on metallurgical processes, several hundreds to thousands of SI were analysed per sample by SEM-EDS (Scanning Electron Microscope coupled with Energy Dispersive Spectroscopy). The position on the cross section of each SI was detected, with special attention being given to those located along welding lines. Major elemental composition analyses of the SI were performed using EDS with a silicon drift detector (SDD) with a large window (about 90 000 cps) coupled with a SEM (FEG JEOL 7001-F) at 15 kV accelerating voltage, and a probe current of about 8 nA. SI were detected by image analysis of the back-scattered electron signal. The Aztec Software (Oxford Company) was used for particle detection and spectra processing.

Trace element composition was then performed on selected SI (about 10 per zone of interest in each artefact) by LA-ICP-MS using the IRAMAT-CEB device (Element XR mass spectrometer from Thermofisher Instrument) according to the methodology published by L’Héritier et al. [32] in order to investigate the geographical provenance of these artefacts. Table 2 describes the main analytical parameters. Comparison of trace element ratios between different iron armatures as proposed by Leroy et al., Disser et al. or L’Héritier et al. [22, 33, 34] can also indicate their common or different area of production. Building site supply, construction phases and ancient metal trade routes can then be explored.The concentrations of 37 elements are routinely determined in SI. Among these elements, up to 14 (Ce, Eu, Gd, Hf, La, Nb, Nd, Pr, Sm, Tb, Th, U, Y, Yb) are used to characterize the geochemical signature of the object. Silicon is measured on the ^28^Si isotope and is used as internal standard by comparison with the SRM610 standard.

**Table 2:** Analytical LA-ICP-MS parameters.

Measured isotopes	Be9, Si28 (internal standard), Ti47, V51, Ni60, Cu63, Ga69, Ge72, As75, Rb85, Sr88, Y89, Zr90, Nb93, Sn118, Cs133, Ba137, La139, Ce140, Pr141, Nd146, Sm147, Eu153, Gd157, Tb159, Dy161, Ho165, Er167, Tm169, Yb172, Lu175, Hf178, Ta181, W182, Pb208, Th232, U238
Laser energy	5 mJ
Laser frequency	10 Hz
Laser spot size	80–100 μm
Uptake (preablation)	10 s
Time analysis	46 s (25 scans of 37 elements)

The compositions of SI cannot be compared directly. SI are composed of a non-reduced part of the ore (slag). As a result, the reduction of iron oxides from the ore into metal would strongly influence SI composition. This enrichment effect can be variable even in the same artefact. This is why SI composition has to be normalized and expressed as ratios or log ratios. As demonstrated in former studies [30], differences in elemental ratio (or log-ratios) are linked to differences in the chemical reduction system (ore, lining, flux adding, charcoal), and cannot be explained solely by the ore composition. As pointed out by Charlton et al. [35], in addition to the influence of the ore, a large proportion of major element content Al, SI, K, Ca can be brought by lining, charcoal or fluxing agent composition, whereas this is less the case for Mn and P whose presence is more closely linked to the initial ore. On the contrary, REE trace elements are most likely contributed by the initial ore. In addition, specific families of SI can be due to the addition of flux during forging. These kinds of SI can be detected when they are located preferentially along welding lines: they were discarded. Other SI with a homogenous composition (similar ratios) can be considered as coming from the same reduction system (i.e. ore, charcoal, lining and flux) used for the ore smelting and can then be assimilated to a given source (or provenance).

For major and trace elements (separately considered), the log-ratio was chosen (pro and cons are discussed elsewhere [34, 36–38]), following the formula:

$$X_{E}=ln(\left[E\right])-\frac{1}{N}\sum_{k=1}^{N}ln(\left[E_{k}\right])$$

where E is the element and X_E_ its log ratio and given that for [E_k_], only the elements well quantified for the whole reference set are considered: Al_2_O_3_, SiO_2_ and CaO (N = 3) for the major elements and Y, La, Ce, Sm and Eu (N = 5) for the traces. The resulting variables were named “Xij”. For the trace elements Xij were calculated for Ce, Eu, Gd, Hf, La, Nb, Nd, Pr, Sm, Tb, Th, U, Y and Yb. For the major compounds, only those which are not reduced during the smelting of the ore were considered (called Non Reduced Compounds—NRC): Al_2_O_3_, SiO_2_, CaO, K_2_O. For a given reduction system as defined by Dillmann and L’Héritier [30] (i.e. ore, charcoal, furnace lining) the respective ratio of these elements (trace and major) should be relatively constant and consequently their Xij too.

Different statistical data treatments were used:

- Hierarchical Clustering Analysis (HCA) on the Xij using the Ward method to discriminate composition clusters;
- for a low dimensional representation of the data, the t-SNE (*t-distributed stochastic neighbor embedding*) algorithm [39] was used (package Rtsne of the R software). The t-SNE algorithm was run with a perplexity of 30. For each dataset, the algorithm was run about 10 times. The result with the lowest Kullback-Leibler divergence (itercost in R package) was chosen.

### Dating

Metallographic observations and chemical analysis of the cross sections are required to support the radiocarbon dating of iron. By revealing the carbon content of the metal and its heterogeneity, they guide the sampling to the most carburized areas (cementite phase Fe_3_C). When possible, two samples were collected in the same cross section. When welding lines and differences in the nature of the iron were observed, several samples were also collected in the different parts of the cross section. The protocol, precisely described by Leroy et al. [10], mainly consisted in drilling with TiN (Titanium Nitride) coated drills of several millimeters in diameter (5 mm) and collecting the iron wires with a magnet. The quantity of metal needed for carbon 14 dating depends on the carbon content in the sample; ideally, 0.5 to 1 mg of carbon is required. The wires were then combusted in sealed tubes with an excess of copper oxide to form carbon dioxide that was finally reduced to a graphite target for the Accelerator Mass Spectrometer (AMS) [40, 41]. The radiocarbon dates were calibrated with the north hemisphere InCal20 calibration curve [42] by using the OxCal software [43].

## Results

### 2.1 Dimensions

**Fig 8:**
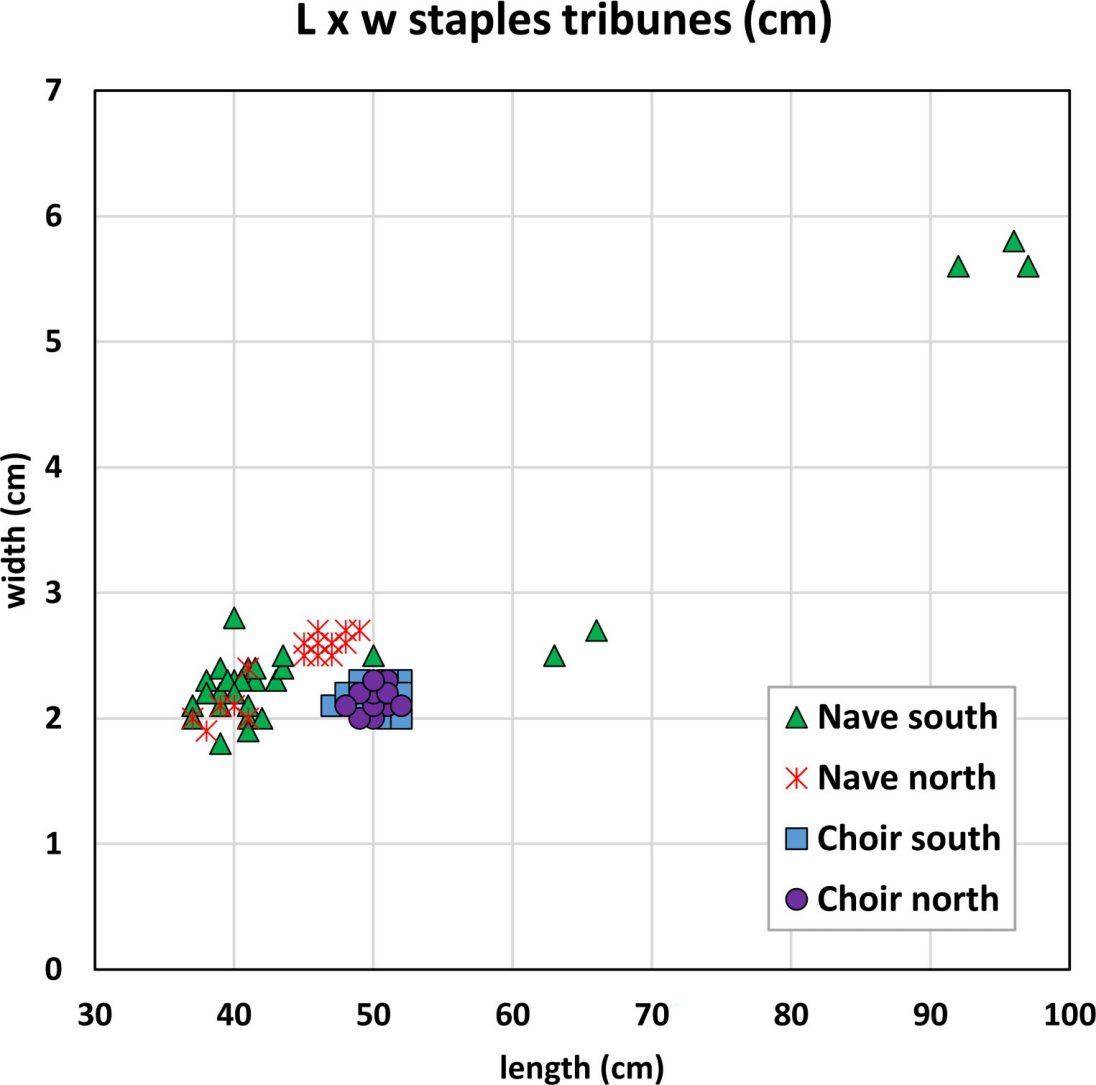
Dimensions of the staples of the tribunes.

**Fig 9:**
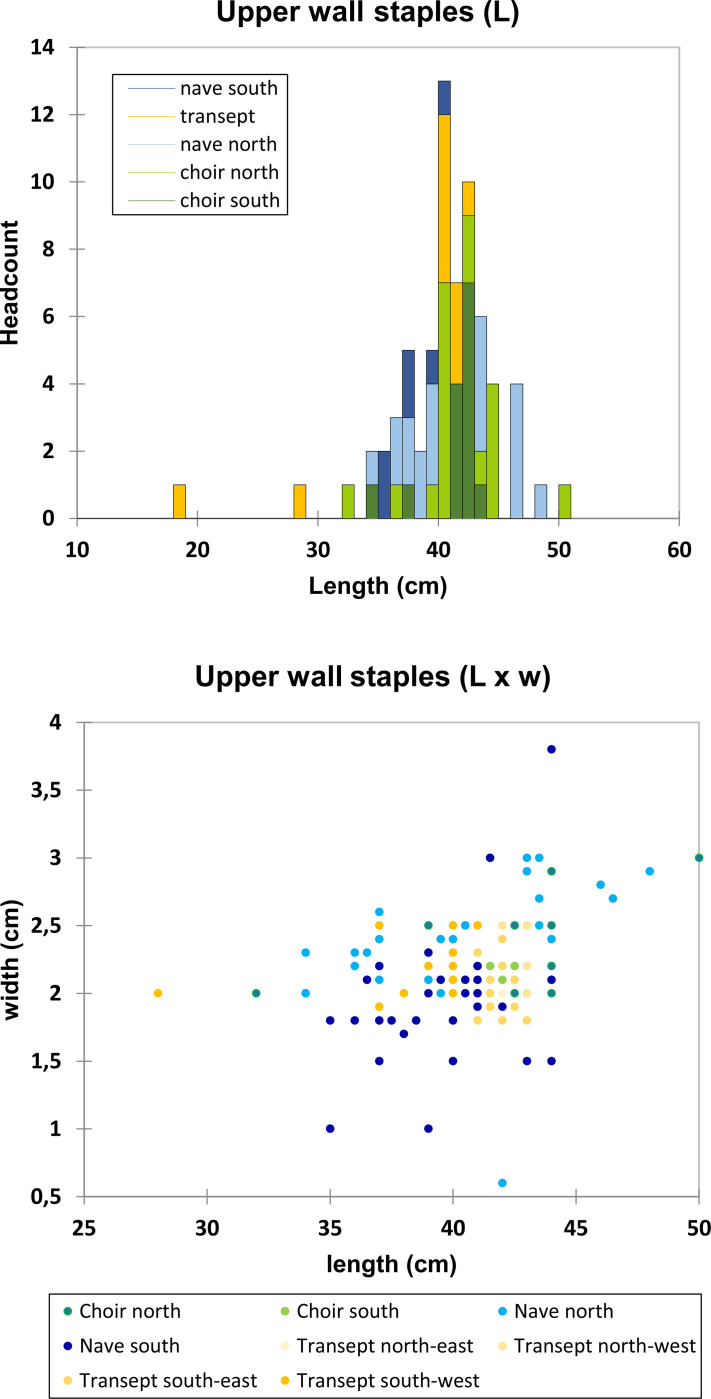
Dimensions of the staples of the upper walls.

The dimensions of the series of staples differ in several ways. Staples attaching the columns (L = 25 cm; w = 2.3 cm; h = 1 cm; m ≈ 300 g) are much smaller than those from the tribunes or the upper walls (L ≈40 to 50 cm, w ≈ 2.5 m ≈ 3 kg), where L is the visible length, excluding the staple legs (Fig 7). However, smaller differences can be highlighted within the two latter groups. In the tribunes, dimensions can clearly be linked to their location (Fig 8): staples from the choir (L = 50 cm, w = 2.2 cm in average, h = 1.5 cm in average) clearly differ from those from the northern (L = 47 cm, w = 2.6 cm, h > 2.5 cm where measurable) and southern parts of the nave (L = 40 cm; w = 2.2 cm, h > 2.5 cm where measurable), except for the last bay of the nave, where the metrics of the north side are coherent with the southern nave. This feature clearly reveals at least three different supplies coming from three different construction phases and suggests that the northern and southern parts of the last bay of the nave may have been erected concomitantly. Seven much bigger staples (L = 50 cm to L = 98 cm, h = 1–1.5 cm) are also found in the southern part of the nave and might originate from one or several restoration phases. On the other hand, staples from the upper walls seem evenly distributed between a 40 to 42 cm long mode without any clear tendency from any part of the building (w = 2.1 cm, h = 3 cm in average) (Fig 9). The legs of the upper wall staples measure 9 cm in average. Depending on their dimensions, the weight of these staples is about 1.5-2kg for the choir tribunes, 2.5–3 kg for the nave tribunes and 3–4 kg for the upper walls.

### 2.2 Nature of the metal

**Fig 10:**
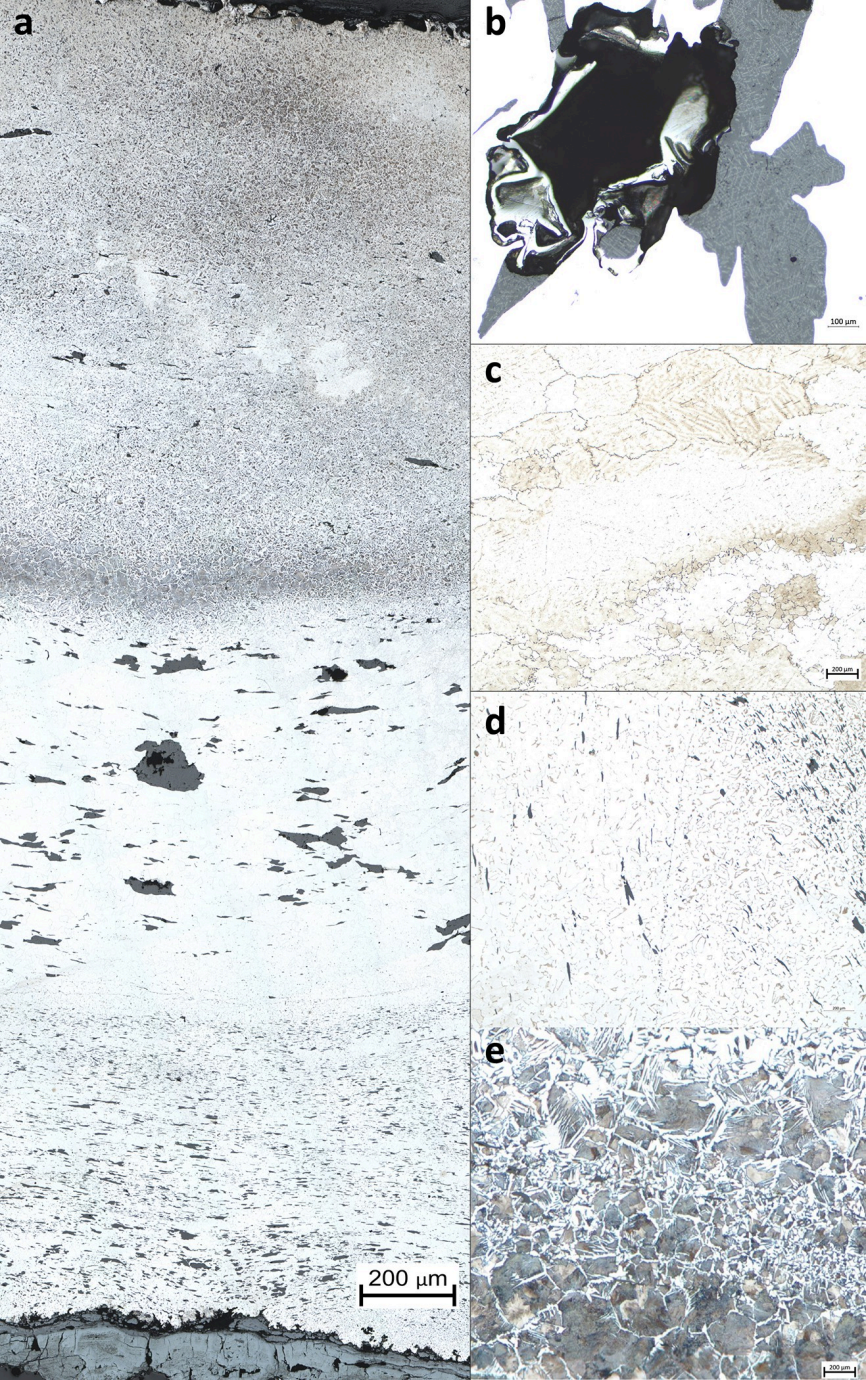
Metallographic features after Nital etching. a. Micrograph of NS8-L showing the welding of ferrous alloys of different qualities (from bottom to top, ferrite with small SI, ferrite with big SI, carburized ferrite-pearlite). b. Slag inclusion and porosity (NS106-T1). C. Ghost structure and different ferrite grain size (NS8-L), d. Slightly carburized zone with Widmanstätten ferrite and small SI (NS106T1) e. Carburized zone with Pearlite and Widmanstätten ferrite (TRIB01S).

The first noticeable feature is that all samples are mainly ferritic, i.e. composed of alpha iron grains with a C content lower than 0.02mass%. In some cases, localized zones showing higher carburization, linked to the presence of ferrite-pearlitic structures, are evidenced (Fig 10). Most of them have less than 15% of their surface with carburized zones. Only NS8-L and TRIB02S have about 40% of their surface made of ferrite-pearlitic zones. However, the global structure of the staples is likely to show a great heterogeneity of composition from one cross-section to another, even if overall they seem to be predominantly ferritic. For example NS8-L is among the most carburized samples and NS8-T sampled on the same staple is almost entirely ferritic. The staples from the tribunes seem slightly more carburized than those from the upper walls. Most carburized staples show Widmanstätten ferrite (Fig 10), which is a common feature in ancient ferrous alloys and testifies a quick (air) cooling of the iron after forging at a temperature corresponding to the austenitic domain.

**Fig 11:**
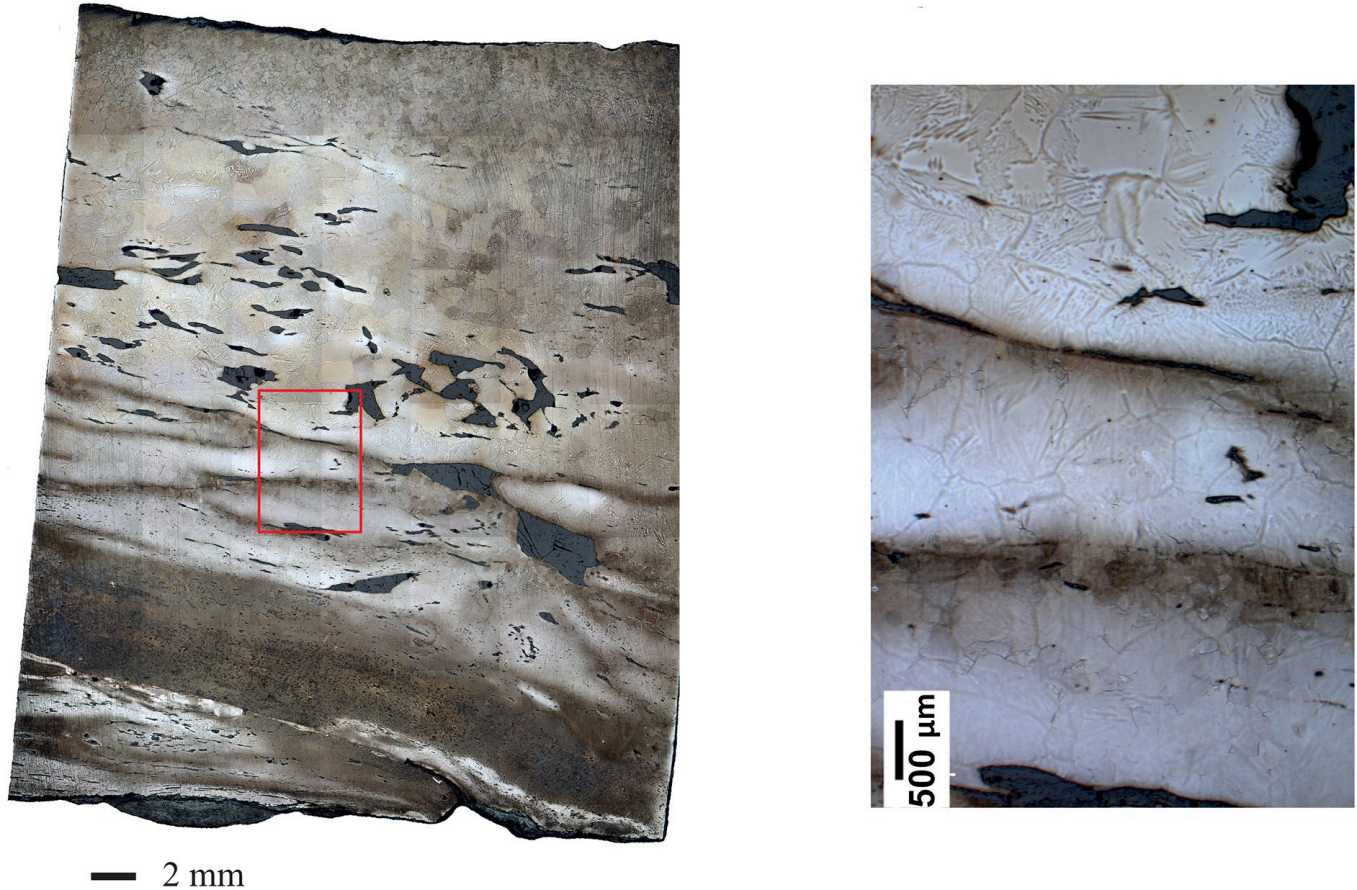
Oberhoffer etching on sample NN9-T showing mesoscopic and microscopic variation in P content (the lighter zones are richer in P).

Ghost structures linked to the presence of phosphorus in solid solution in variable contents [44] are also visible on several samples (staples NN1, NN9, NS8, VN4, TRIB01S and TRIB02N) (Fig 10). This high variation in phosphorus is also confirmed by Oberhoffer etchings on samples from staples NN1, NN9, and VN4 (Fig 11).

All cross sections reveal the presence of non-metallic SI in varying proportions, from 1.5% to 7% of the surface of the metallic matrix (Fig 10). Such quantities of SI are frequent in ancient ferrous alloys especially those used for construction purposes [45]. Whereas some cross sections mostly contain small SI, other disclose many SI measuring more than 5 mm long by 1 mm wide. This is particularly the case for cross sections coming from staples NN1, NN9 and NS106 that all clearly show numerous large millimeter sized SI in their structure (Fig 11). In some samples, porosities (holes in the metallic matrix) are also observed, revealing that the compacting of the iron bloom was not perfect (Fig 10).

**Fig 12:**
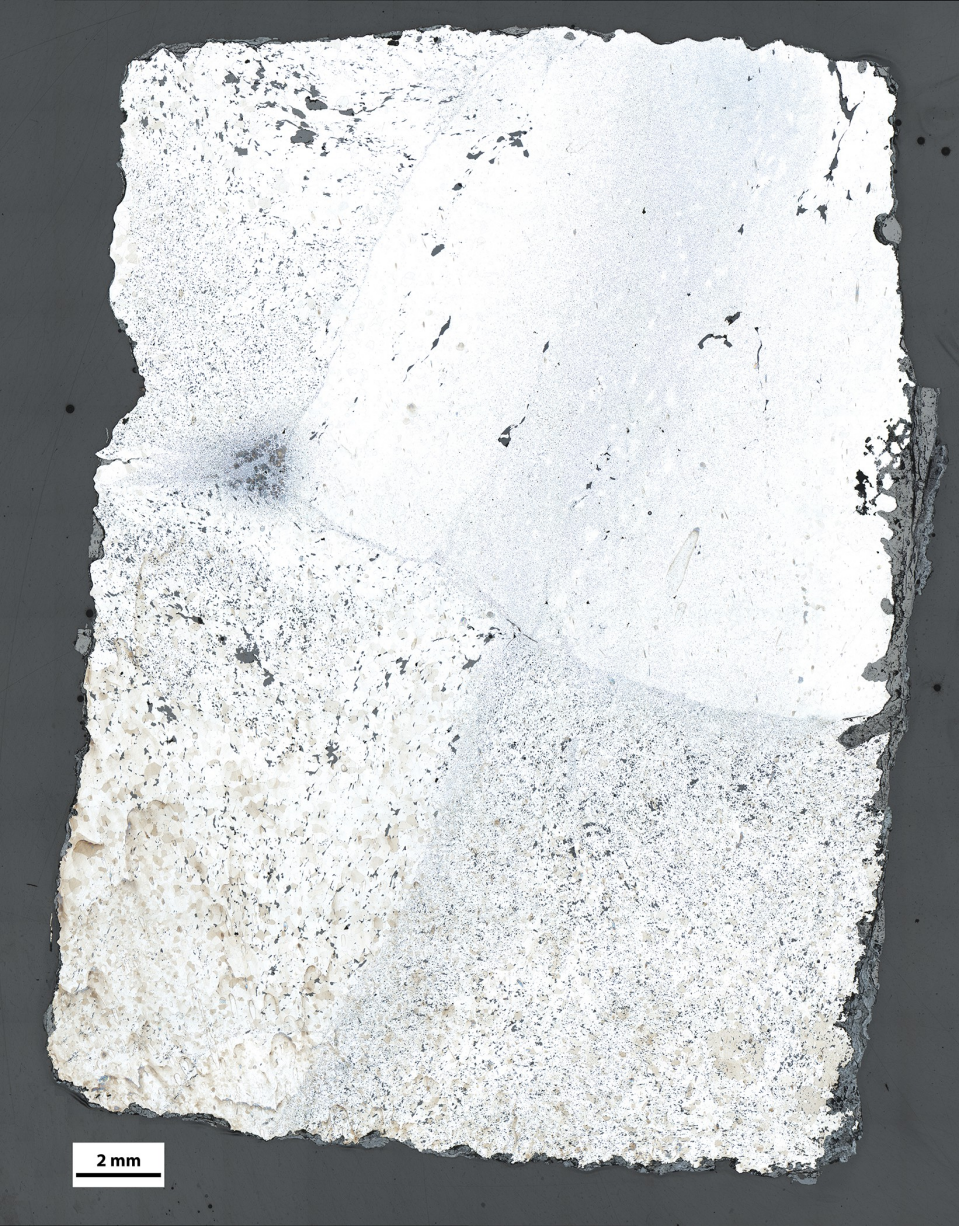
Micrograph of sample VN2-T showing several welding lines and different microstructures (Nital etching).

One original feature of the studied corpus is that at least one cross section and sometimes several per staple present clear welding marks inside their microstructure (Fig 12). This was observed for all staples except TS01. Moreover, on both sides of these welding lines, the microstructure often shows very different features regarding grain size, SI size and numbers or carburization. This suggests that at least 10 staples out of 12 were forged by welding at least two different pieces of iron together. Some samples such as GUA01, TRIB01S and TRIB02N show only one welding line, but others, particularly in the upper wall staples, show a succession of 2 or even up to 4 superposed welding lines. While these welding lines were mostly observed on cross-sections sampled in the middle of the staples, the examples of NS8T and NS8L also show that they can be present both in the middle and at the end of the staple.

Table 3. sums up all these results concerning metallographic analyses.

**Table 3:** Results of metallographic analyses.

	% of SI (area)	Numerous large SI	% carburized zones (area)	Ghost structures	Widmanstätten ferrite	Number of welding lines
**CH1-L**	1.5%	No	0%	no	-	0
**CH1-L2**	3.2%	No	22%	no	yes	0
**CH1-T**	4.7%	No	< 1%	no	yes	1
**GUA 01**	2.4%	No	4%	no	yes	1
**NN1-L**	4.0%	Yes	0%	yes	-	2
**NN1-L2**	3.2%	Yes	< 1%	yes	no	1
**NN1-T**	5.9%	Yes	0%	yes	-	0
**NN9-L**	7.0%	Yes	14%	yes	no	4
**NN9-L2**	8.5%	Yes	4%	Yes	-	2
**NN9-T**	4.1%	Yes	6%	yes	no	1
**NS106-T**	5.5%	Yes	32%	no	yes	1
**NS106-T2**	4.7%	Yes	0%	no	-	0
**NS7-T**	2.0%	No	16%	?	yes	2
**NS8-L**	5.9%	No	36%	yes	yes	2
**NS8-T**	2.6%	Yes	1%	no	yes	2
**TRIB01S**	3.0%	No	31%	yes	yes	1
**TRIB02N**	3.9%	No	40%	yes	yes	1
**TS01-T1**	1.7%	No	14%	no	yes	0
**TS01-T2**	4.1%	Yes	3%	no	yes	0
**VN2-T**	6.5%	No	2%	no	yes	3
**VN4-L**	2.5%	No	7%	yes	no	2
**VN4-L2**	2.4%	No	6%	yes	yes	0
**VN4-T**	2.0%	No	0%	yes	-	1

### 2.3 Dating of the staples

**Fig 13:**
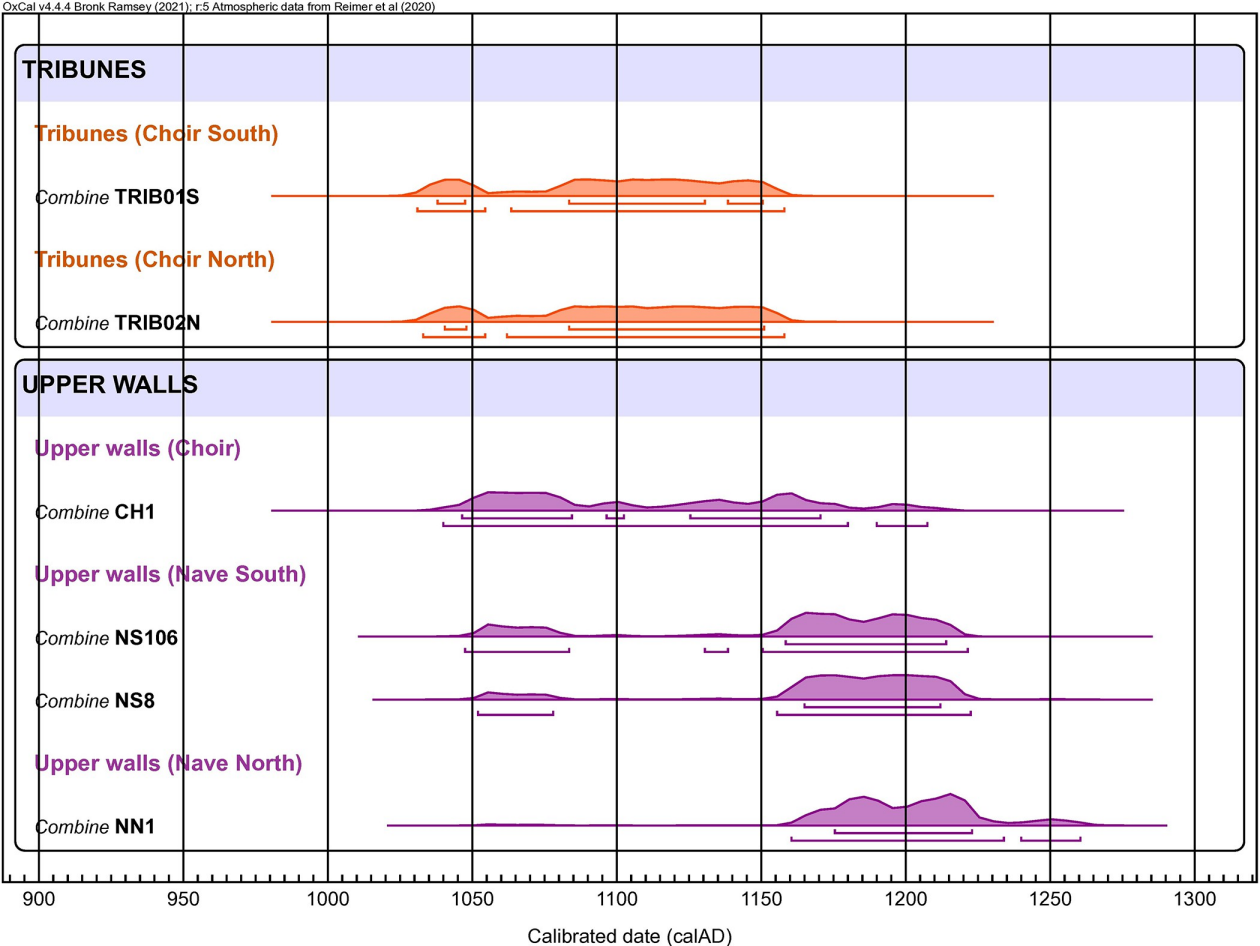
Comparison of radiocarbon dating of the staples of the tribunes and the upper walls.

In the most carburized areas of six staples, local sampling of metal was performed for radiocarbon dating. Two of the dated staples come from the tribunes and four from the top walls. Two or three ^14^C measurements were obtained per sample and their good consistency allowed us to group them with the R_Combine operation of the OxCal program to give a more accurate dating (Table 4 and Fig 13). The results show that all samples are contemporaneous with the construction phases of Notre-Dame in the middle of the 12^th^ or beginning of the 13^th^ century, thus indicating that they were probably produced and implemented coeval to the erection of the cathedral. The results also show that both samples from the tribunes are somewhat older than those from the top walls. Despite two slightly different dates obtained on staple CH1, the combination of both dates seems coherent with this pattern. Other samples should however be analyzed on this part of the building for a better resolution. The date combinations show that the tribune samples are not later than the 1160s whereas the top wall samples can be possibly later than the 1200s, suggesting approximately a 50 years shift.

**Table 4:** Results of radiocarbon dating on the staples of Notre-Dame de Paris.

Sample	Sub sample	Lab. ID	Extracted carbon content (mg)	pMC	Radiocarbon Age (BP)	Calibrated age (cal AD) (2F073, 95.4%)*
**Tribunes (choir south) staples**						
TRIB01S	TRIB 1S_1a	SacA 61087	0,42	89.32 ± 0.24	905 ± 30	1041–1108 (38.7%) 1115–1216 (56.8%)
	TRIB 1S_1b	SacA 61088	0,85	88.81 ± 0.25	955 ± 30	1026–1160 (95.4%)
	TRIB 1S_2	SacA 61091	0,73	88.25 ± 0.23	1005 ± 30	990–1050 (63.6%) 1080–1154 (31.8%)
	*Combine TRIB 1S (Chi2 test T = 5*.*6<6)*				955 ± 18	1030–1054 (18.0%) 1063–1158 (77.5%)
**Tribunes (choir north) staples**						
TRIB02N	TRIB 2N_1a	SacA 61089	0,98	88.69 ± 0.23	965 ± 30	1024–1158 (95.4%)
	TRIB 2N_1b	SacA 61090	1,04	88.57 ± 0.25	975 ± 30	998–1002 (1.0%) 1020–1158 (94.5%)
	TRIB 2N_2	SacA 61092	0,46	89.3 ± 0.24	910 ± 30	1040–1214 (95.4%)
	*Combine TRIB 2N (Chi2 test T = 2*.*7<6)*				950 ± 18	1032–1054 (16.2%) 1062–1158 (79.3%)
**Upper walls (choir) staples **						
CH1	CH1a	SacA 63209	0,53	88.72 ± 0.27	960 ± 30	1025–1160 (95.4%)
	CH1b	SacA 63210	0,62	89.71 ± 0.25	870 ± 30	1047–1083 (11.1%) 1130–1138 (0.8%) 1150–1261 (83.5%)
*Combine CH1 (Chi2 test failed T = 4*.*5>3*.*8)*					915 ± 22	1040–1180 (89.7%) 1190–1207 (5.8%)
**Upper walls (nave south) staples **						
NS106	NS106a	SacA 64728	0,35	90.02 ± 0.29	845 ± 30	1159–1266 (95.4%)
	NS106b	SacA 64729	0,57	89.06 ± 0.25	930 ± 30	1032–1178 (93.2%) 1192–1202 (2.3%)
	* Combine NS106 (Chi2 test failed*, *T = 4>3*.*8)*				888 ± 22	1047–1083 (18.2%) 1130–1138 (1.1%) 1150–1221 (76.1%)
NS8	NS8b	SacA 63212	0,39	89.47 ± 0.26	895 ± 30	1042–1106 (30.5%) 1118–1220 (64.9%)
	NS8c	SacA 63213	0,43	89.86 ± 0.27	860 ± 30	1052–1077 (5.8%) 1156–1262 (89.6%)
	* Combine NS8 (Chi2 test T = 0*.*7<3*.*8)*				878 ± 22	1052–1078 (8.8%) 1155–1222 (86.7%)
**Upper walls (nave north) staples**						
NN1	NN1a	SacA 64724	0,87	89.7 ± 0.25	875 ± 30	1046–1084 (13.8%) 1096–1102 (0.6%) 1125–1230 (79.2%) 1242–1258 (1.8%)
	NN1b	SacA 64725	0,68	90.17 ± 0.27	830 ± 30	1166–1269 (95.4%)
	* Combine NN1 (Chi2 test T = 1*.*1<3*.*8)*				853 ± 22	1160–1234 (89.4%) 1240–1260 (6.1%)

* OxCal v4.4.4 Bronk Ramsey (2021); r:5 Atmospheric data from Reimer et al. (2020)

### 2.4 Composition of SI

#### Major element composition

**Fig 14:**
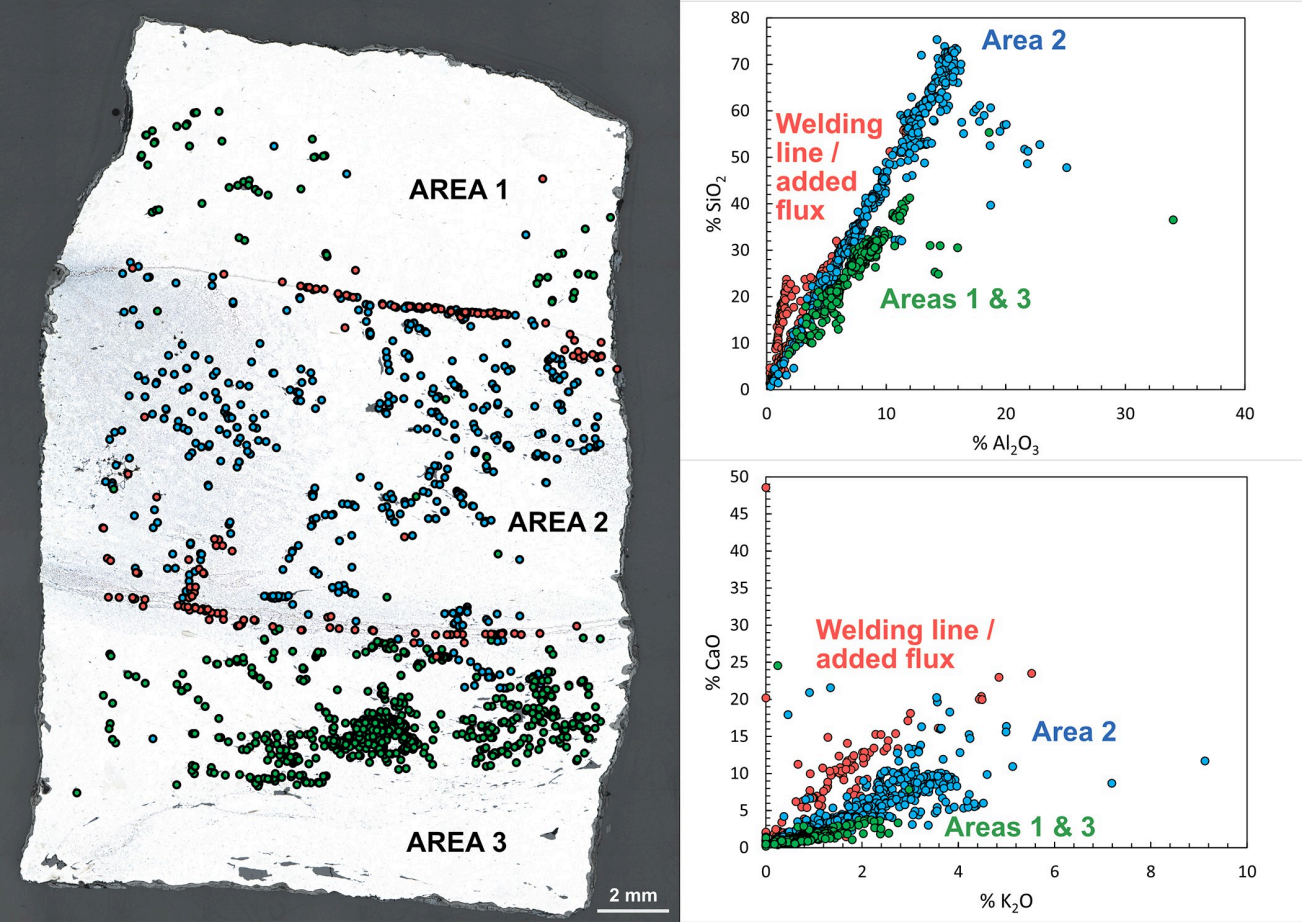
SI composition regarding their location in sample NS7-T. Composition clusters (blue, green, red) were determined by HCA on Xij data. The clusters were then plotted on binary graphs highlighting differences in Al_2_O_3_/SiO_2_ and K_2_O/CaO ratios between the clusters.

As far as major elements are concerned (results available in S1 Table), apart from GUA01 and NN9, all samples with welding lines exhibit different clusters of inclusions (linked to different compositional ratios) located in different areas of the transverse section separated by welding lines. As illustrated in Fig 14 for NS7, this is the case for TRIB01S, TRIB02S, NS7, NS8, NS106, VN2 and maybe also NN1 (Table 5). In some samples such as NS7 or NS8 where two parallel welding lines were observed, delimiting three superimposed zones, SI composition of the central zone (Area 2) is different from the upper (Area 1) and lower (Area 3) zones (Fig 14). This suggests that to forge the staple using two different bars, one of them was spilt in two and the other was inserted in the middle and then hot-forged as in a cleft weld. Moreover, the use of SiO_2_-rich (sand) or CaO-rich flux in welding lines is also evidenced in this case (Fig 14). Other staples also show distinct NRC ratios in several samples. This is the case for VN4 and CH1 for which no welding line was observed on the different samples. However, the weld of different materials is unveiled by the difference in SI composition. Only one artefact (TS01) displays a single NRC ratio, even if several cross-sections were analyzed. To sum up, only one staple (TS01) has a homogeneous SI composition with no evidence of welding, two (NN9 and GUA01) have homogeneous SI but show one or several welding lines and the nine remaining staples have different SI on either side of welding lines (or on different samples). This assembly of iron coming from different ore-lining-charcoal reduction systems (or workshops) highlighted by the differences in SI composition for 9 out of 12 artefacts [30] has up to now usually been associated with recycled iron (i.e. recycling of scrap iron by welding) [46, 47].

**Table 5:** Different clusters highlighted by welding lines and major elements analyses and variation in P_2_O_5_ and MnO content (given in average weighted content per cluster).

Staple	Number of SI clusters	P_2_O_5_*	MnO*
GUA01	1	2.4%	1.3%
CH1	2	< 0.5%	< 0.5%
NN1	2	< 0.5%-1.1%	< 0.5%-1.1%
NS7	2 and flux welding	1.4–1.8%	0.9–1.2%
VN2	2	0.7%	< 0.5–2.7%
NN9	1	1.2%	0.7%
NS106	3 or more	1.1%	< 0.5–0.7%
NS8	2 or 3	1–1.3%	0.6–1.2%
TS01	1 ?	0.9%	< 0.5%
VN4	4	3.7–5.3%	0.6–1.5%
TRIB01S	2	2.1–3.1%	< 0.5%
TRIB02N	3 or 4	1.7–3.2%	< 0.5%

SI have small yet detectable contents in P_2_O_5_. Only VN4 has around 5% in P_2_O_5_. TRIB01S, TRIB02N and GUA01 have around 2–3% and other samples are usually below 2%. On the other hand, MnO is often below detection limits: only GUA01, VN4, NS7, NS8 and NN9 have about 0.5–1% Mn0 and VN2 has a particular cluster with up to 2.7% MnO. This already suggests the use of ores of different compositions and/or origins.

### Trace element composition

**Fig 15:**
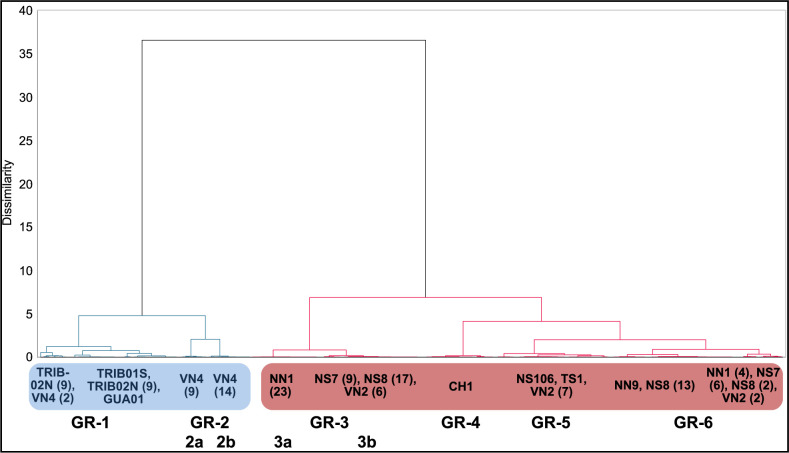
HCA on the SI trace element composition (Xij) of all samples. The two main branches with the highest dissimilarity are highlighted in blue and red. For samples represented in different branches, the number of SI in each branch is indicated in brackets. Clusters were determined by the elbow method.

**Fig 16:**
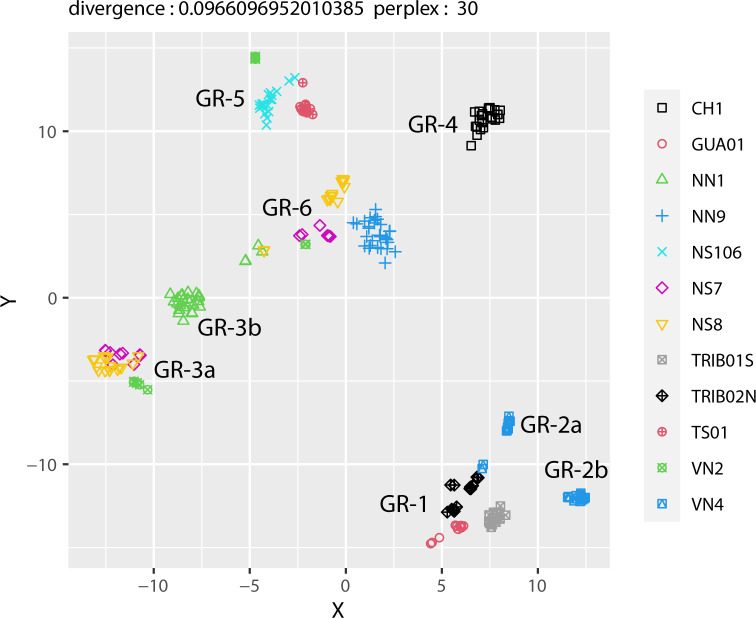
t-SNE on the SI trace element composition (Xij) of all samples.

Trace element composition of SI (results available in S2 Table) shows a slightly different pattern from that of the major elements (Table 6). Whereas most samples clearly exhibited two different major element NRC ratio clusters, especially on both sides of a welding line, this is much less the case regarding trace elements. Despite their welds and/or heterogeneities in major element composition, the SI of GUA01, TRIB01S, TRIB02N, NS106, NN9 and CH1 are grouped in single trace element clusters as shown by the HCA and t-SNE (Figs 15 & 16). Each of these staples was therefore probably forged from one or several pieces of iron coming from a single ore source. This is also the case for TS01 whose SI are homogeneous in major and trace elements and in which no weld was visible. The pattern is however less clear for NN1 whose SI are distributed in two different groups, both in HCA and t-SNE (although one group is largely predominant). On the other hand, HCA and t-SNE both show that VN2, VN4, NS7 and NS8 are each made of pieces of iron coming from at least two different supply sources (three for VN2 and maybe also for NS8). Therefore, one third to half the iron artefacts indicate mixes of provenances (use of different ore sources), which has so far usually been associated with recycled iron and confirms the results obtained with major element analyses [46].

**Table 6:** Summary of provenance results. “?” means unsure compatibility as very few SI are involved in the group or staples belong to different subgroups (a&b).

Staple	Number of iron sources (major elements)	Number of iron sources (trace elements)	Provenance group
CH1	2	1	GR-4
NN1	2	1 maybe 2	GR-3a, GR-6 (?)
NN9	1	1	GR-6
NS106	3 or more	1	GR-5
NS7	2 + flux	2	GR-3b, GR-6
NS8	2 maybe 3	2 maybe 3	GR-3b, GR-6
VN2	2	3	GR-3b, GR-5, GR-6 (?)
VN4	4	2 maybe 3	GR-2a&b, GR-1 (?)
TRIB01S	2	1	GR-1
TRIB02N	3 or 4	1	GR-1
GUA01	1	1	GR-1
TS01	1 ?	1	GR-5

Considering the 12 artefacts altogether, HCA points out two very dissimilar clusters, highlighted in red and blue on Fig 15. These two clusters are also clearly visible on the t-SNE plot (“blue” HCA cluster isolated on the bottom right and “red” HCA cluster on the top left). The first one (blue) comprises all lower level staples (both tribune staples TRIB01S and TRIB02N and the nave aisle staple GUA01) and one staple from the upper walls (VN4), whereas the second one (red) only includes staples from the upper walls. This first dissimilarity suggests that very different iron sources were used for these two clusters. However, both plots also reveal that each of these two clusters is far from homogeneous. Within the first one (blue cluster), two subgroups can be distinguished: the first one (GR-1) comprises the lower level staples TRIB01S, TRIB02N and GUA01 as well as 2 SI from VN4, whose other SI are scattered in a second less homogeneous subgroup (GR-2a & 2b). Within the second one (red cluster), at least four different subgroups can be distinguished. CH1 seems totally isolated, forming a subgroup on its own (GR-4), which is coherent with the staple location in the choir. TS01 and NS106, which are both closer to the southwestern angle of the transept crossing, may also share the same origin as they are grouped in the same sub-branch in the HCA plot and are close on the t-SNE plot, with a few SI of VN2 (GR-5). Lastly, both plots reveal interesting information for the rest of the upper wall staples, located in the middle of the nave. Indeed, the SI of NS7, NS8 and VN2, three staples that were implemented side by side on the southern side of the top walls of the nave, are split in the same two subgroups (GR-3 & GR-6) and therefore seem to share two different origins. One of these sources (GR-3a) could be shared with NN1 (GR-3b), a staple located opposite on the northern side. The other source (GR-6), which is maybe less homogeneous, involves another northern staple, NN9, and a few SI of NN1.

Overall, at least six and up to eight different clusters are evidenced by SI analysis. Clusters that are sharply separated (both in HCA and t-SNE) clearly correspond to different iron sources, some staples even belonging to several sources. The most heterogeneous artefacts seem to be located in the middle bays on the southern part of the nave, where all four studied staples are made of at least two, sometimes three different metal pieces (VN2, VN4, NS7, NS8). Moreover, whereas VN4 has specific provenances, on the other hand, the SI of NS7, NS8 and VN2 are mixed on t-SNE and HCA diagrams and share several common origins, indicating that iron pieces from the same supply sources were used to forge different staples, which were probably placed during the same construction campaign. The SI of other staples are also relatively close on the t-SNE and HCA diagrams. For example in GR-6, NN1 and NN9 could possibly share the same origin as NS7 and NS8.

## Discussion

These results provide unprecedented information on the use of iron in Gothic construction as well as on the iron trade and ironwork in the High Middle Ages.

### 3.1 Use of iron in early Gothic constructions

Archaeological observations and radiocarbon dating performed on the iron staples sealed on the floor of the tribunes proves that the builders implemented them in the earliest phases of construction, most probably in the early 1160s. So far, these series of staples are the earliest known example of iron armatures used in the initial design of a Gothic monument. They were designed probably 15 to 20 years earlier than the tie-rods installed in Soissons cathedral [48] southern transept and about 40 years before the iron reinforcements of Chartres or Bourges cathedral, which until now were considered as the first examples of a systematic use of iron in such masonries [10, 15, 16, 22]. These staples are placed on the floor of the tribunes, on the line corresponding to the extrados of the arches between the outer and inner ambulatory, as well as on the transverse arches of the outer ambulatory. Let us recall that the elevation of the building began with its outer shell, precisely where these staples were used [49]. This metal grid, installed during the first construction phases, should be interpreted as the innovative reinforcement of the cross-ribs of the outer ambulatory peaking at almost 11 m high, which had to be maintained without any internal support and without the weight of the upper floors for a certain time. Whereas other buildings used wooden tie rods stretched between the arches and therefore visible as in Laon, Chartres, Soissons, Amiens, Reims, Tours, or Beauvais cathedrals [20, article chaînage, 50–53] that were eventually cut, or later on in the 13^th^ century iron tie rods that were finally removed (as in Soissons, Reims or Beauvais [13, 17]), the first master builder of Notre-Dame de Paris made the bold choice of a system using a more durable material that could be more easily concealed. The same system was used in the bays of the nave, whose construction followed that of the choir, with the exception of the most western bay, which was built later. Dating of the nave staples could not be performed here, but the system resemblance suggests a continuity in construction techniques, even if different craftsmen were called upon, leading to slightly different sizes for the batches of staples. According to the study of the mouldings and careful measurement of architectural members, Bruzelius argues that a second architect was in charge roughly from 1170 to 1190 and started the construction of the nave whilst finishing the upper parts of the choir [49]. We should therefore assume that this second architect adopted the construction techniques of the first master. What seems certain is that the easternmost bays of both sides of the nave (north and south) were built successively: the size difference of the staples clearly indicates that they belong to two different batches, such observations confirming Bruzelius’ hypotheses [49].

Our results also reveal that the 13^th^-century architects likewise chose to use iron staples as reinforcements. The rows of staples implemented at the top of the great lateral walls of the building were dated from the early 13^th^ century at the latest. Dating is however not precise enough for the sole choir staple dated to propose a phasing between the upper nave and upper choir. Yet, the dates obtained are in good agreement with the dating of the 13^th^ century framework (1215 AD for the nave and 1225 AD for the choir [54]), indicating that the erection of the wall and the installation of the staples occurred shortly before the completion of the framework.

The dating of these staples located at the top of the walls clearly shows the master mason’s decision to prevent the shearing forces resulting from the lateral thrusts of the framework. According to Viollet-le-Duc’s comments in his *Dictionary of architecture*, the same type of reinforcement seems to have been already used at the end of the 12^th^ century in the checked cornice of the choir (Fig 3), which supported the first framework of the cathedral erected in 1185 [20, tome 2, article chaînage]. Although dating could not be performed here as these staples are nowadays not visible or available for study (the question remains open if Viollet-le-Duc had them removed or not when he had the great iron chains and tie-rods installed), these reinforcements could well be the work of the second architect. The first Gothic frames, heirs of the Romanesque period, are indeed made of a repetition of trusses where the oblique pieces—the rafters—formed the load-bearing structure of the frame, with only the main trusses reinforced by horizontal tie beams [55]. Such an arrangement generates large lateral thrusts, which are only partially contained by the framework tie-beams [56]. The masonry reinforcement process, probably developed in the late 12^th^ century by the second architect, was taken up in the support of the 13^th^ century frameworks, despite its higher slope which limited the lateral thrusts and its better longitudinal bracing [56, 57]. This continuity in techniques, from the lower level of the tribunes, to the top of the building and probably involving at least three master masons over a 50-year timespan is striking in Notre-Dame. Its master builders decided to employ forms known since Antiquity [58, 59], such staples being for example extensively used in the Colosseum in Rome [60], in a novel implementation to serve an innovative architecture. It is worth noting that a similar iron reinforcement system can also be found in the collegial church of Mantes-Le-Jolie, whose framework was built in the same decade as Notre-Dame in the beginning of the 13^th^ c., showing the spread of these construction techniques between neighboring building yards, probably partly involving the same craftsmen [61]. Later, in the middle of the 13^th^ c., master builders adopted instead the solution of real chain-links embedded at the top of the walls, as in the basilica of Saint-Denis or in the Sainte-Chapelle [62, 63].

### 3.2 Iron quality and forging

The nature of the ferrous alloys (mainly ferritic and partly phosphorous, with a great deal of embedded SI) used to make these reinforcements is quite common for the Middle Ages. A similar quality was already found in Troyes, Chartres, Metz, Beauvais, Rouen or Auxerre cathedrals for example [23, 31, 45, 64]. The most striking feature about Notre-Dame’s staples is however the number of welding lines that can be observed in their microstructures, indicating that several pieces of iron, sometimes from different provenances, were welded together to form each staple. This is the case for the upper wall staples, but also for that of the tribunes, despite the 40- to 50-year gap between these two groups.

One must first consider that, in the field of construction iron research [12], the study of Notre-Dame is almost the only one where entire staples were sampled and analyzed by metallography, especially for the 12^th^ and 13^th^ centuries. Other examples with access to entire bars are Avignon and Metz dating from the 14^th^ and 15^th^ century [31, 65] and yet, in these two cases, usually only one sample was crosscut from each bar, ruling out the possibility of focusing systematically on such a feature. This difference in sampling might be the first reason why such a great number of welds were observed in the present study. Let us also add that all the welds of Notre-Dame were only discovered through metallographic and microscopic examinations, whereas the great bars in several buildings (Saint-Denis, Bourges, Reims…), weighing from 20 to 50 kg, have to be forged from different pieces of iron, resulting in clearly visible marks on the artefacts [15, 66, 67]. For smaller elements, such an implementation has so far been described as evidence of recycling, especially when the welding of metal from different workshops or different origins is observed as in Notre-Dame, where 9 samples out of 12 show clear differences in SI major element composition out of which 6 reveal different provenances with trace element analyses [47, 68]. A first possibility could therefore be that almost all the iron used in the building yard from the middle of the 12^th^ century to the early 13^th^ century came from hot forging and the assembling of scrap metal. This would mean however that the proportion of recycling (up to 9 staples out of 12) was much higher than what has been observed so far in other monuments, where only 10 to 30% of iron seemed to come from this practice [47]. Moreover, these previous studies showed that recycling tended to be favoured for the forging of small elements weighing a few hundred grams rather than for bars of several kg. It could also involve multiple folding of some flattened metal pieces in order to shape them with a rectangular or squared cross section. Comparison with archaeological finds in different contexts also shows this tendency to recycle scrap iron from multiple welding of flattened metal pieces [69–71]. Reuse and recycling was indeed a very common practice in the Middle Ages for all construction materials, for various purposes [72–75]. Regarding metal, economic issues have so far always prevailed, especially in renovation phases where older armatures can be reused or reshaped into the new structure [47]. However, we are not dealing here with a renovation. Furthermore, in a rich building yard such as Notre-Dame with maybe twice to four times more financial resources than other building sites [76], such a predominance of recycling throughout the entire construction appears questionable. Other hypotheses should therefore be proposed.

It is very likely that the capacities of iron production at that time made it possible to forge a 2–3 kg staple from a single bloom. Surveys on archaeological semi-products show that since Antiquity, iron blooms (i.e. the crude mass of iron produced in a smelting furnace) could largely exceed 10 to 15 kg and that bars up to 5 kg could well be produced using a single bloom or a bloom fraction [66, 77]. Iron units from 1.7 to 5 kg were common in ancient forms of transport [78]. The welds observed in the staples are therefore not likely to be linked to limitations in iron production conditions. However, a majority of the upper wall staples such as NS7, NS8, VN2, VN4, NN1 and NN9 clearly show a median weld, indicating that the staples were forged using two 1.5 to 2 kg bars joined together at one end using a cleft weld. What is the root cause of these welds? What constraint led the blacksmith to assemble two smaller bars instead of using a single slightly bigger one? Interestingly, the SI composition of NS7 and NS8 (and VN2) reveals that these two (or three) bars which were placed next to each other in the masonry and probably forged by the same blacksmith also probably share the same two origins for at least one of the primary pieces of iron they were forged with. This could well indicate that the smith had at his disposal (in his workshop or in the yard itself), 1.5 to 2 kg bars coming from at least two different supplies and used them indistinctively to forge bigger 3 to 4 kg staples. In the tribunes, the welding process appears to be a little different (only one welding line is visible indicating no cleft weld but rather a straight scarf one) but could be linked to the same practice, with smaller 1 kg bars. In either case, this suggests that the need to perform welding was linked to the size of the bars supplying the building site (or the blacksmiths themselves). Another argument to support this hypothesis is the dating: despite the differences in origins and the welding lines, there seems to be no great difference in date between the two parts of a single staple. Thus, they may well be from different but contemporary productions, sharing a common trade route rather than being involved in a recycling practice. It is well known for earlier and later periods that bars of different sizes were commonly traded. The shape and size of these bars could correspond to different material qualities or serve to identify a precise producer [78–82]. For the 15^th^ c. in Roussillon (South of France), Verna [81] mentions the existence of iron in *verga*, in *cairo* or in *plata*, each of them possibly having different sizes: e.g. verga of 10 units per quintal (42 kg) so approx. 4.2 kg, and others of 14 units per quintal (approx. 3 kg). Sometimes the weight of these pieces is not mentioned, suggesting that a common unit weight existed. All the sizes mentioned here are at least two to three times bigger than the 1 to 2 kg bars of Notre-Dame but it should be remembered that towards the end of the Middle Ages, hydraulic forges were fully developed in the area, enabling larger bars to be more easily forged than was possible manually [82, 83]. What is also striking is that there is a great permanency in the forging practices over more than 50 years. We therefore put forward the hypothesis that this almost systematic use of welds to forge the staples is probably linked to the building site supply and perhaps also to the transport conditions (or to the providing bloomeries). The iron unit available at the forge seems to be no larger than 1 to 2 kg.

Another hypothesis can be suggested: the welds could come from the use of bigger bars (made of smaller blooms) that were cut to forge the staples. Archaeological evidence of the transport of iron bars by ship supports the transport of such great iron bars, which were sometimes forged from smaller pieces [66, 78, 84]. In the case of Notre-Dame, this hypothesis is supported by one particular feature observed on staple NS8, which presents a weld at the end of one of its legs (NS8-L). This weld does not seem useful in the process of forging the staple and might therefore rather originate from the iron bar that was used by the smith. However, this feature is not systematic, as samples from NN9, VN4 and CH1 cut on the legs of the staples do not show any weld. Although there is currently a lack of evidence to give full credence to this hypothesis, it cannot be ruled out. The analysis of a greater number of staples (and especially staples sampled side by side like NS7 and NS8) would be necessary to investigate this point.

### 3.3 Provenance

**Fig 17:**
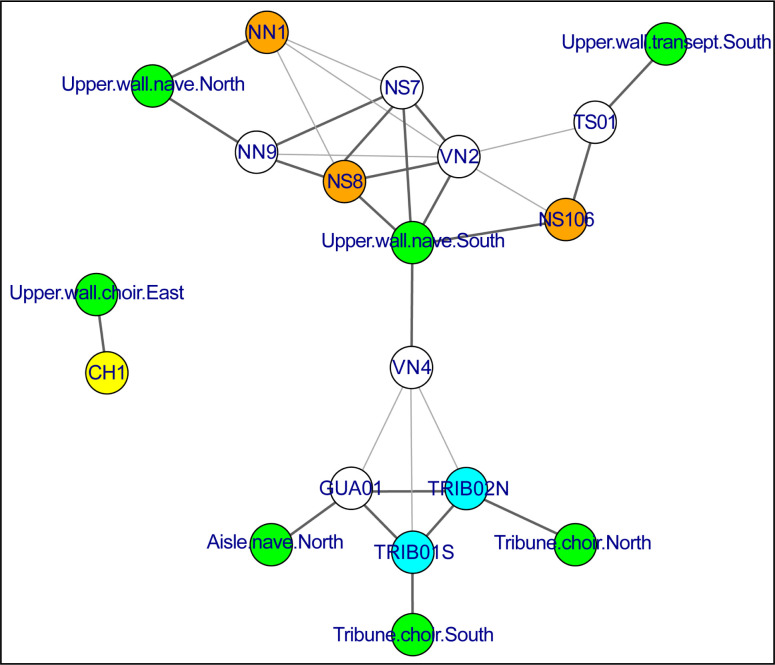
Network graph indicating the relationships between the staples. Orange: 95% probability beg. of the 13^th^ c., Cyan: 95% probability middle of the 12^th^ c. Yellow: 95% probability middle of 12^th^, beg. of 13^th^ c., Green: location. When two staples are linked, they show at least one constitutive part with the same provenance (see Table 6). Thick lines indicate certain common provenance. Thin lines indicate unsure common provenance (noted “?” in Table 6). Each staple is also linked to its location.

At least six different provenances were identified thanks to SI trace element analysis. Moreover, no predominant iron source seems to prevail. This feature is once again very specific to the case of Notre-Dame. Most of the building yards studied so far such as Brussels, Bourges, Metz or Rouen, showed some consistency in the provenance groups [22, 29, 33, 85]. The relatively small number of staples studied and their scattering over the whole building could be a reason for this great diversity (Fig 17). For example, the specific provenance of CH1 (GR-4), which is the only staple studied in the apse, may seem quite logical. NN1 and NN9, which are located in the same bay however seem to come from different sources. Nonetheless, two interesting matches can be highlighted. The first one is on the middle bays of the upper nave: on the southern side NS7, NS8 and VN2 all set in the same bay share two identical origins (GR-3 & GR-6), maybe corresponding to the same supply phase. Each origin is shared with one of the staples from the northern bays (NN1 & NN9). The second match concerns TS01 and NS106, which are on both sides of the southwestern corner of the transept and could also share the same provenance (GR-5). Therefore, there seems to be some consistency in the iron supply pattern of the building yard, despite a few outliers. This pattern would need to be investigated by studying a greater number of staples. The great diversity in the possible provenances could also be a reflection of the intensity of the iron market in Paris, whether the supply sources were local or more distant. Let us recall that iron used to be traded both ways along the Seine River in the late Middle Ages [86] mainly coming from the Marne but also downstream from Normandy. The proximity of the Seine and the renowned port of Saint-Landry on the northeastern banks of the Île de la Cité, next to Notre-Dame’s building yard made it considerably easier to transport materials [87]. Iron supply by land transportation was also likely: tax exemptions for the bishop’s carts are mentioned in the 12^th^ and 13^th^ century archives [6].

Another interesting feature is the chronological evolution in iron supply: the upper wall staples (with the possible exception of VN4) come from different sources to the staples from the tribunes and nave aisle next to the Guadalupe chapel. In the earliest phases of construction (mid and late 12^th^ c.), only one iron source (GR-1) has been identified so far whereas, in the early 13^th^ century, supply relies on at least five other sources (GR-2 to GR-6), four of which (GR-3 to GR-6) have radically different chemical signatures. This means that between the construction of the lower parts of the cathedral in the 1160s (choir) and 1180-1200s (nave) and the erection of the upper walls in the early 13^th^ century, there was a major change in iron supply sources for Notre-Dame’s building yard, with the emergence of several new iron sources supplanting the former ones. Could this be linked to changes in the management and financing of the construction? It is well known that Bishop Maurice de Sully was the initiator and commissioner of the construction of Notre-Dame in the second half of the 12^th^ century, but what was the exact role of the chapter? The canons appear to have played a major role in the cathedral’s construction, maybe from the earliest construction stages and especially after Maurice de Sully’s death in 1196 [87]. Did iron supply partly rely on the Bishop’s or the chapter’s domains? On another level, major changes also occurred at that time in Paris, which became a true capital city during the reign of Philippe Auguste (1180–1223) where bourgeois and merchants played a essential role from the late 1190s. Could the drastic shift observed in iron supply be linked to these social changes and a new organization in the supply of the city and/or the building yard? And where did the welding and mixing of iron from different provenances take place: in the blacksmith’s workshop or on the building yard, or prior to that, before the iron reached Paris? All these hypotheses and questions could be explored with the analysis of additional staples and crossing these data with slag analyses from identified archaeological iron production sites to link the iron armature of Notre-Dame with regional or more distant bloomeries.

## Perspectives & conclusions

The study of iron reinforcements used in the construction of Notre-Dame de Paris offers a glimpse into the innovation that took place on the building site of Notre-Dame as well as the permanency of construction techniques throughout the construction campaigns, adapting metal to create a novel architecture. Notre-Dame is now unquestionably the first known Gothic cathedral where iron was massively used to bind stones as a proper construction material. Now that the presence of iron armatures dating from the different construction periods of the cathedrals has been highlighted, the function of these staples in the cathedral structure can be studied, in relation with the general structure of the building and the mechanical behaviour of all its materials. Beyond the obvious need to use such iron implements given the medieval builders’ conception of their architecture, what role did these staples play (and do they still play today) in the cathedral’s equilibrium?

Moreover, this study also renews our understanding of the circulation, trade and forging of iron in the 12^th^ and 13^th^ century capital of the French kingdom. The discovery of numerous welds and multiple provenances thanks to a metallographic study and SI chemical analyses sheds light on the activity of the iron market in a major medieval European city and on the nature of the goods that circulated. These results need to be reinforced with the opening of the Notre-Dame restoration yard and accessibility to new structures thanks to the dismantling of stone and repair of the framework, but also by implementing a comprehensive database of regional iron productions to better trace its circulation.

## Supporting information

S1 Table Results of SI analyses by SEM-EDS (in %). (XLSX)

S2 Table Results of SI analyses by LA-ICP-MS (in ppm, unless Si in %). (XLSX)

S1 Fig Nital etched micrographs of staple CH1. (TIF)

S2 Fig Nital etched micrograph of staple GUA01. (TIF)

S3 Fig Nital etched micrographs of staple NN1. (TIF)

S4 Fig Nital etched micrographs of staple NN9. (TIF)

S5 Fig Nital etched micrograph of staple NS7. (TIF)

S6 Fig Nital etched micrographs of staple NS8. (TIF)

S7 Fig Nital etched micrographs of staple NS106. (TIF)

S8 Fig Nital etched micrograph of staple TRIB01S. (TIFF)

S9 Fig Nital etched micrograph of staple TRIB02N. (TIF)

S10 Fig Nital etched micrographs of staple TS01. (TIF)

S11 Fig Nital etched micrograph of staple VN2. (TIF)

S12 Fig Nital etched micrographs of staple VN4. (TIF)
